# Genetic architecture of basal heart rate and its modulation by dietary salt in *Drosophila melanogaster*

**DOI:** 10.1371/journal.pgen.1012313

**Published:** 2026-10-05

**Authors:** Gulnur Ipek Erdemli, Murat Yilmaz, Alp Mete Ummet, Fatih Akdemir, Ergi Deniz Ozsoy

**Affiliations:** 1 Faculty of Science, Department of Biology, Functional and Evolutionary Genetics Laboratory, Hacettepe University, Ankara, Türkiye; 2 Hacettepe University the Institute for Graduate Studies of Science and Engineering, Ankara, Türkiye; 3 Institute for Human Genetics and Department of Genetics and Biochemistry, Clemson University, Greenwood, South Carolina, United States of America; 4 Atatürk University, Department of Medical Biology, Faculty of Medicine, Erzurum, Türkiye; Universidad de Valparaiso, CHILE

## Abstract

Heart rate (HR) is a complex quantitative phenotype that is influenced by both genetic and environmental factors, and it is a critical factor in the regulation of cardiac physiology. Despite the discovery of numerous genes associated with HR variation in humans and other organisms, the mechanisms by which these genes interact to affect HR changes in natural environments and their responses to environmental stressors remain incompletely understood. In this study, the genetic basis of basal HR and the response to salt stress were examined using the *Drosophila* Genetic Reference Panel (DGRP). The 154 DGRP lines exhibited moderate broad-sense heritability and substantial genetic variation in HR measurements during the third larval stage, with males and females analyzed separately, under both standard and 0.1% NaCl-containing medium conditions. The mixed-effects model revealed that the interaction between genotype and environment significantly influences HR. Genome-wide association analysis identified gene variants associated with critical biological processes such as ion transport, cardiac development, protein homeostasis, metabolism, and stress response. The identification of different genes under standard and saline (NaCl-supplemented) conditions suggests that environmental stress unlocks cryptic genetic variation. Candidate genes were tested using heart-specific GAL4 drivers in RNAi experiments, and a considerable number of these genes were found to significantly affect HR based on sex and the environment. These findings reveal that HR variation is strongly shaped by genotype-by-environment interactions and sex-specific genetic effects, with distinct sets of genes contributing under basal and stress conditions. The identification of environment-dependent genetic architectures suggests that salt stress can expose cryptic genetic variation influencing cardiac function. By integrating genome-wide association analyses with targeted RNAi-based functional validation, this study provides a framework for linking natural genetic variation to physiological phenotypes in a context-dependent manner.

## Author summary

HR is a vital physiological characteristic for life and an important marker for detecting heart disease. This marker is a trait influenced by both genetic factors and environmental factors, but the underlying genetic mechanisms and how they interact with environmental conditions are not well understood. In this study, we investigated the genetic architecture of HR using natural genetic variation in the fruit fly *Drosophila melanogaster*. By analyzing genotype × environment interactions under dietary salt treatment, we demonstrated how environmental stress reveals hidden genetic variation. Functional experiments demonstrate that HR is regulated by genetic networks associated with ion homeostasis and cellular regulatory mechanisms. Our findings indicate that the genetic architecture of this complex trait is responsive to the environmental context and modulated by evolutionarily conserved mechanisms.

## Introduction

Cardiovascular diseases are among the leading causes of death worldwide. According to data from the World Health Organization (WHO), these diseases accounted for 32% of all deaths in 2019, with ischemic heart disease accounting for approximately 13% of global deaths [1,2]. Cardiovascular diseases arise from functional disorders of the heart and the associated vascular systems. Stroke and ischemic heart disease are the most prevalent examples of this category of diseases [3]. Vascular structural abnormalities and physiological parameters, including heart rate (HR), are critical in the pathogenesis of these diseases [4–6]. As an integrated indicator of cardiac performance, autonomic balance, ion-channel activity, and vascular tone, HR serves as an early biomarker that reflects the physiological state of the organism. An elevation in resting HR increases myocardial oxygen demand while shortening the diastolic filling time. This condition makes people more likely to have ischemia and makes atherosclerotic plaques more fragile by putting more stress on the blood flow [4]. Meta-analyses have shown that a 10-bpm increase in HR is associated with a 15–20% increase in the risk of cardiovascular disease and all-cause mortality [7,8]. However, the clinical effects of HR may vary depending on the context: in some cases, it may be a risk factor, whereas in other clinical conditions it may have protective properties. In this regard, studies based on large datasets and clinical cohorts indicate that a high resting HR, depending on genetic background, may be a risk factor in diseases such as dilated cardiomyopathy, but that this increase in HR may potentially exert protective effects in conditions such as atrial fibrillation or recurrent ischemic stroke [9–11]. A low HR increases the stroke volume and may contribute to structural changes in the atrium and causes structural abnormalities, thereby increasing the risk of arrhythmia. Atrial fibrillation is one of the primary causes of thrombus formation in the heart. As a result of the reduced risk of atrial fibrillation, a high HR indirectly reduces the risk of stroke [10]. Genome-wide studies have shown that HR is a polygenic trait influenced by numerous genes, with associated genes showing strong expression patterns particularly in cardiomyocytes [10,12]. Nevertheless, its genome-wide genetic architecture, how this variation is regulated under environmental stressors, and the conditions under which HR becomes a reliable predictor of cardiovascular risk remain poorly understood [9,12,13]. This highlights the need for a systematic examination of the polygenic basis of HR and its sensitivity to environmental factors from both biological and clinical perspectives.

The *Drosophila* model is important for studying HR and cardiac physiology in adult and larval stages, enabling the investigation of cardiovascular diseases and congenital heart defects in humans, as well as related stress factors [14,15]. *Drosophila* is a great model for studying the genetic basis of cardiac phenotypes because many of the genes that control heart development, contraction mechanisms (cardiomyocytes), and ion channel function in *Drosophila* are structurally and functionally similar to their human orthologs [14–17]. In this context, it has long been known that the evolutionarily conserved transcription factors *Hand* and *Mef2* are important for *Drosophila* heart development before and after eclosion. Specifically, *Hand* has been shown to be important for cardioblast differentiation and heart morphogenesis in the early stages of cardiogenesis. *Mef2*, on the other hand, is important for the differentiation and maturation of cardiac muscle cells [18,19]. The fact that these gene functions are the same in different species supports the idea that studying *Drosophila* heart development is a powerful and versatile approach to investigating human heart biology.

*Drosophila melanogaster* is a flexible model organism for studying the genetic basis of complex phenotypes and diseases in humans. This is due to its evolutionarily conserved genome, approximately 75% of human disease-related genes have orthologs in *Drosophila*. *Drosophila melanogaster* also has advanced genetic tools such as RNAi and the UAS/GAL4 system. Its short life cycle, low maintenance costs, and ability to rapidly produce large sample sizes enable practical and reproducible physiological studies to be conducted; these characteristics have facilitated their widespread use in HR and cardiac physiology studies over the past three decades [20–22]. Despite having a single-chambered heart structure and an open circulatory system, *Drosophila melanogaster* is a functionally suitable model organism for cardiac physiology studies due to its evolutionarily highly conserved heart physiology, even when compared to model organisms such as *Danio rerio* and *Mus musculus* [21,23,24]. The genetic and physiological adaptability of *Drosophila* enables the examination of cardiac function as well as the impact of environmental stressors, such as salt consumption, on the cardiovascular system [25].

The health implications of a high salt diet have been a topic of debate for a long time, and it is particularly linked to elevated blood pressure [26,27]. Various cardiovascular diseases, particularly hypertension, as well as metabolic and systemic disorders, are induced by excessive salt consumption, which disrupts ion balance [28–30]. Similar to humans, the homeostasis of ions such as Na^+^, Cl^−^, and H^+^ is critical for the normal functioning of systems such as the central nervous system, digestive system, respiratory tract, and urinary system in *Drosophila* [29–31]. While dietary salt has been shown to influence processes such as nutrition and learning in *Drosophila* [25], and a few studies have addressed its effects on adult cardiac function and cardiac aging - typically through targeted, candidate-gene approaches in a limited number of genetic backgrounds [32] - the genetic basis of the HR response to salt has not been examined across natural genetic variation. In particular, no genome-wide screen using a natural-variation panel has yet addressed how genetic background modulates salt-induced changes in HR. In this context, the impact of dietary salt on the genetic determinants of HR, a vital physiological parameter of heart health, remains largely unknown. This indicates that there must be a systematic investigation of the effects of salt on cardiac physiology in relation to the genetic background [33].

In this context, the evolutionarily conserved cardiac biology of *Drosophila melanogaster*, combined with its powerful tissue-specific genetic toolkit, provides a robust in vivo model for dissecting the polygenic basis of HR and its responses to environmental influences. Building on this, we used the *Drosophila* Genetic Reference Panel (DGRP), a collection of inbred lines with fully sequenced genomes [34,35], to investigate the genetic architecture of basal HR and its modulation by dietary salt. We quantified HR in third instar (L3) larvae, analyzed separately for males and females, reared on either standard medium or a 0.1% NaCl diet, allowing us to assess both natural genetic variation in basal HR and its interaction with salt intake, a recognized cardiovascular risk factor. We then combined genome-wide association analyses, to identify candidate genes associated with HR regulation [15,36,37], with tissue-specific RNAi to test the functional contribution of these candidates in cardiac tissue.

Because the *Drosophila* heart undergoes extensive structural and functional remodeling during metamorphosis including changes in innervation, contractile architecture, and flow dynamics [15,38] we focused on the L3 larval stage, which provides a relatively stable, predominantly myogenic system that resembles the intrinsic myogenic regulation of vertebrate and mammalian hearts [15,39]. To our knowledge, this is the first systematic analysis of the genetic basis of basal larval HR and its response to dietary salt across a broad panel of natural genetic variation, thereby addressing an important gap in the literature.

## Results

To investigate the genetic architecture of HR and its modulation by environmental conditions, we analyzed HR measurements obtained from 154 DGRP lines reared in standard and salt-supplemented media. Measurements were taken during the third larval stage (L3); males and females were analyzed separately, and 20 larvae were used for each line × treatment × sex combination. HR was quantified from video recordings by counting the number of contractions over a fixed 20-second observation period for each larva.

We first characterized phenotypic variation across lines and conditions using descriptive statistics. To quantify the relative contributions of genetic and environmental factors, we applied variance-partitioning approaches, including analysis of variance and mixed-effects models, allowing us to evaluate the effects of genotype (Line), treatment, sex, and their interactions. In addition, genotype-by-environment interactions were assessed to determine how different genotypes respond to salt exposure. Line-specific differences between environments were further evaluated using Welch’s t-tests with multiple testing correction.

To identify genetic variants associated with HR variation, we performed genome-wide association analyses using mean HR values across lines, sexes, and treatment conditions. Functional interpretation of candidate genes was conducted using gene set enrichment analysis. Finally, selected candidate genes were experimentally validated using tissue-specific RNAi approaches, and their effects on HR were assessed using appropriate statistical comparisons.

### Quantitative genetic variation in heart rate

To characterize the extent of phenotypic variation and to assess the contribution of genetic and environmental factors, we first analyzed HR across 154 DGRP lines reared under standard and salt-supplemented medium conditions.

DGRP lines are ordered independently within each sex based on increasing mean HR under control conditions to facilitate visualization of genotype-specific responses to salt exposure relative to their baseline levels. This representation highlights how the magnitude and direction of salt-induced changes vary across genetic backgrounds, reflecting genotype-by-environment (G × E) interactions. Accordingly, line positions are not directly comparable between panels, and each panel should be interpreted independently.

We first used descriptive statistics to examine the distribution and range of HR values across lines and conditions. An examination of descriptive statistics for larval HRs in lines reared on a standard medium revealed that the lowest and highest mean HR values (beats per 20 s) for females were 51.4 and 143.7, while the corresponding values for males were observed as 50.2 and 140.9 (Fig 1A and 1B, mean values are provided in S1B Table). Likewise, for larvae reared on salt-supplemented media, the minimum and maximum mean HRs (beats per 20 s) were recorded as 50.2 and 185.0 in females, compared to 52.1 and 188.1 in males (Fig 1A and 1B, S1B Table). To determine if the differences observed among the lines were statistically significant and attributable to genetic variations, we conducted separate analyses of variance (ANOVA) for each treatment and sex. This analysis revealed that the line term was highly significant in all cases (p < 0.001, S2A Table), indicating substantial genetic variation underlying HR. A collective assessment of broad-sense heritability estimates for standard and salt-supplemented media indicates that larval HR is a trait with moderate broad-sense heritability in both sexes (females *H*^*2*^ = 0.45, males *H*^*2*^ = 0.47). Notably, the contribution of the genetic component was observed to increase relatively in both sexes under salt stress conditions (*H*^*2*^ = 0.51, S2A Table). These results suggest that environmental stress makes genotypic differences in HR relatively more discernible. To further investigate this pattern, we next analyzed the combined dataset to assess genotype-by-environment interactions.

**Fig 1:**
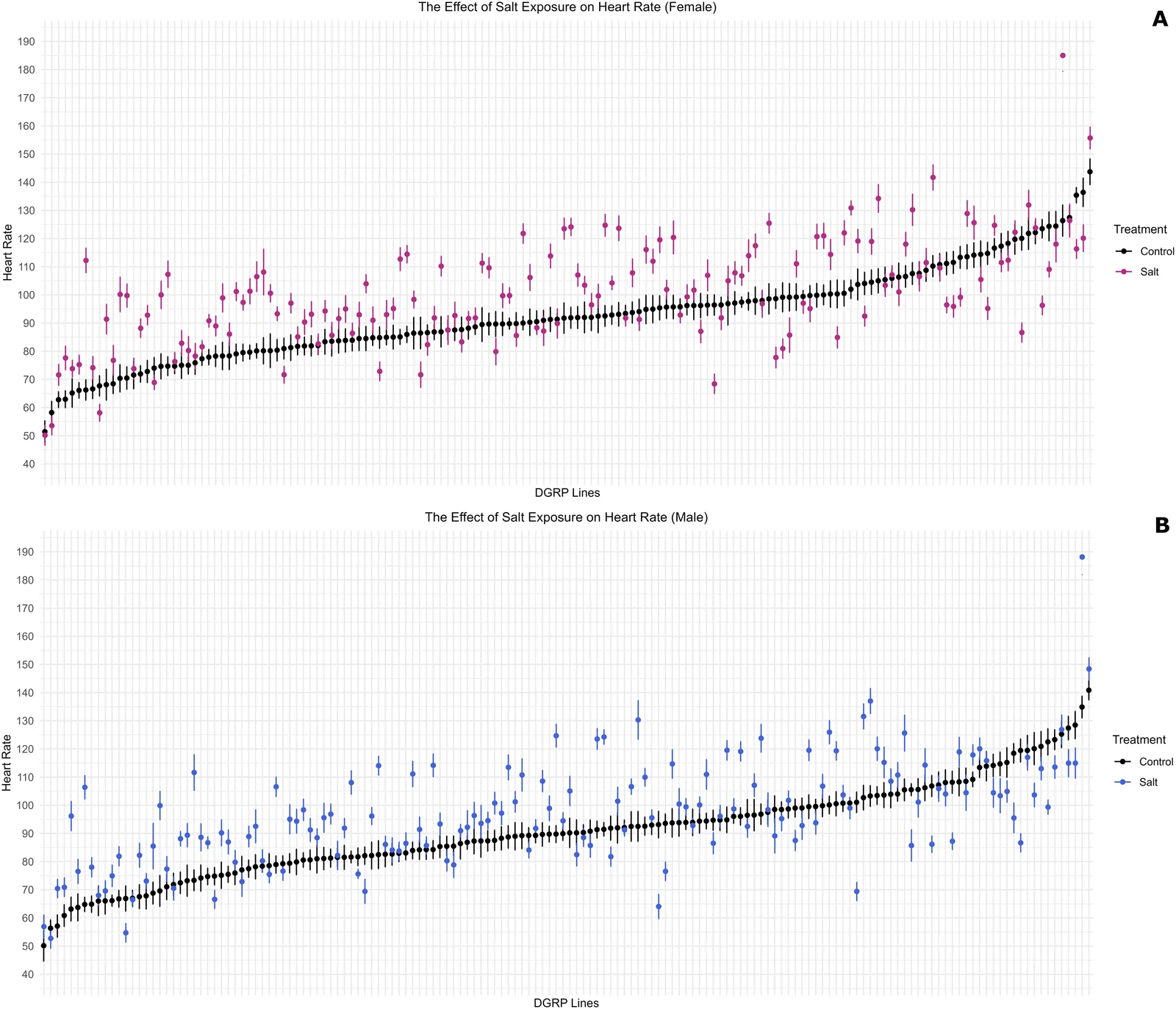
Variation in HR across DGRP lines under control and salt conditions. Mean HR (beats per 20 seconds) measured in third instar (L3) larvae across 154 *Drosophila* Genetic Reference Panel (DGRP) lines under control (standard medium) and salt-supplemented (0.1% NaCl) conditions. **(A)** Female larvae. **(B)** Male larvae. Each point represents the mean HR for a given DGRP line, and vertical bars indicate the standard error of the mean (SEM). Black points correspond to control conditions (standard medium), while colored points indicate salt treatment (pink for females, blue for males).

To jointly evaluate the effects of genotype, environment, and sex, we applied a mixed-effects model to the combined dataset. This analysis indicated that larval HR varies significantly with respect to both the fixed effects of treatment and sex, as well as their interaction. Analysis of the fixed effects revealed that both treatment and sex terms are highly statistically significant (p < 0.001, S2A Table). The treatment-by-sex interaction, while weaker, was statistically significant (p = 0.046, S2A Table), suggesting that although the responses of females and males to environmental variables are not identical, they do not exhibit substantial differences. Importantly, this model also allowed us to quantify genotype-by-environment (GxE) interactions through the line × treatment interaction term. As indicated by the analysis results, the genotype-by-environment (line-by-treatment) interaction term for the HR phenotype is statistically highly significant and accounts for a substantial portion of the observed variance; in other words, larvae exhibit differential HR responses depending on the environment in which they develop. This pattern is further illustrated by reaction norm plots (S1A and S1B Fig), where each line represents the response of a genotype across environments. Variation in the slopes and direction among lines indicates heterogeneous genotype-specific responses to salt exposure, providing a visual representation of the observed genotype-by-environment interaction.

To further interpret the biological implications of this interaction, we examined the consistency of line-specific responses across environments by correlating the mean heart rate of each line (per sex) between standard and salt-supplemented media. The results point to a moderate cross-environment correlation for the HR phenotype in L3 larvae of both sexes. Correlation coefficients were found to be r = 0.65 for both sexes, indicating a highly significant relationship (p < 0.001, S2A and S2B Fig). While this moderate correlation suggests partial conservation of genetic effects across environments, the deviation from unity (r ≠ 1) indicates that genotypes do not respond uniformly, supporting the presence of genotype-specific environmental responses (i.e., G × E).

To complement the mixed-model results and identify genotypes contributing to the observed genotype-by-environment interaction, we performed separate Welch’s t-tests for each line to compare HR between larvae reared on standard and salt-supplemented media, analyzing female and male larvae separately with P-values adjusted for multiple testing using the Benjamini–Hochberg false discovery rate procedure (S1B Table). For females, the t-test results indicated that a significant number of the examined lines (60 lines) exhibited statistically significant differences in HR between the two treatments. Among the lines showing significant effects, the salt treatment increased HR in 48 lines, whereas in 12 lines it led to a decrease in HR (Fig 2A). Multiple t-test analyses performed on male larvae indicated a similar pattern as seen in females. A total of 46 lines exhibited statistically significant variations in HR between larvae developed on the standard medium and those on the salt-supplemented media; among these lines, HR increased in 37 lines and decreased in 9 (Fig 2B).

**Fig 2:**
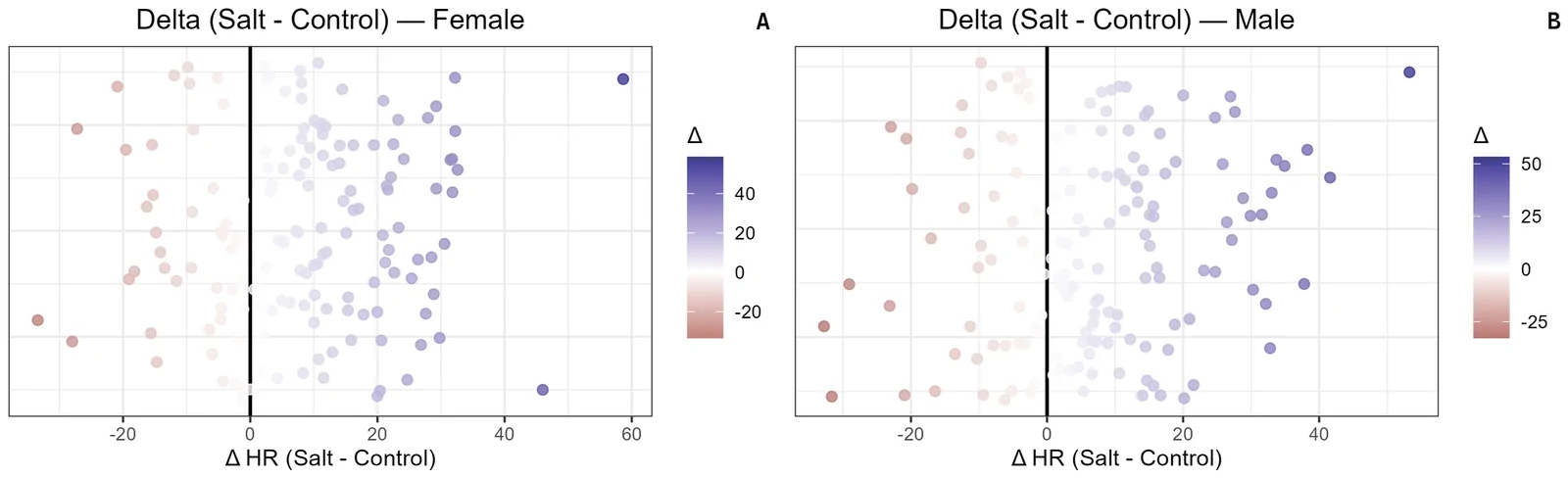
Salt-induced changes in heart rate (ΔHR). Scatter plots show the difference in heart rate between salt-supplemented and control conditions (ΔHR = Salt − Control) across DGRP lines. Unlike Fig 1, which displays absolute HR under each condition, Fig 2 directly visualizes the genotype-by-environment (G×E) interaction: by expressing each line’s salt response as a single, baseline-independent value (ΔHR), it reveals how genotypes differ in both the direction and magnitude of their response to salt, making the line-specific heterogeneity that constitutes the G×E effect immediately apparent. The vertical line indicates ΔHR = 0. Positive values represent an increase in heart rate under salt stress, whereas negative values indicate a decrease relative to control conditions. (A) Female larvae. (B) Male larvae. Color intensity reflects the magnitude of ΔHR. Statistical significance for individual line-specific responses was evaluated separately using Welch’s t-tests; therefore, the figure primarily represents descriptive ΔHR distributions across DGRP lines rather than statistical significance for individual lines.

These results indicate that the overall genotype-by-environment interaction detected by the mixed model reflects heterogeneous, line-specific changes in HR, as illustrated by the distribution of ΔHR values across genotypes (Fig 2).

### GWA analysis of heart rate and gene set enrichment analyses

Genome-wide association (GWA) analyses were performed on the average HRs of larvae obtained from two different rearing conditions in DGRP lines to map candidate variants regulating HR. These analyses were conducted considering males, females, and sex averages and differences. GWAS analyses were performed using a linear mixed model (LMM) framework implemented in FaST-LMM, incorporating a genomic relatedness matrix (GRM) to control for confounding due to genetic relatedness among lines. This approach is particularly appropriate for DGRP-based analyses, as the panel exhibits limited global population structure and rapid linkage disequilibrium decay, thereby enabling relatively high-resolution genotype–phenotype mapping [34]. Polymorphic inversions, polygenic relatedness effects, and *Wolbachia* infection were all considered to improve the analysis’s reliability [35]. Given the relatively small genome size of *Drosophila* and the reduced multiple testing burden compared to human GWAS, a threshold of p < 10^−5^ was used to identify candidate variants. This threshold is consistent with previous DGRP studies, where similar uncorrected significance levels have been used to detect variants-often of low frequency-with potentially large phenotypic effects, rather than relying on highly stringent multiple testing corrections.

The genome-wide association analysis identified 44 candidate variants and 35 candidate genes associated with HR in L3 larvae raised in the control culture medium (p < 10^−5^), and 56 candidate variants and 39 genes associated with HR in larvae reared in the salt-supplemented culture medium. Analyses of HR differences between the two rearing conditions revealed 34 candidate variants and 30 genes. In total, three GWAS analyses identified 134 candidate variants associated with the HR phenotype, of which 101 were uniquely associated with gene regions. The identity analysis of variants located in gene regions revealed 101 single nucleotide polymorphisms (SNPs), 8 deletions, 2 insertions, and 1 multiple nucleotide polymorphism (MNP) (A complete list of candidate variants and their genomic annotations is provided in S3A–S3D Table).

When the distribution of variants was examined according to gene regions, it was discovered that the majority were found in intronic (91 variants) and downstream (22 variants) regions. In other regions, 11 variants were found in the upstream region, 7 in the 3’UTR region, 2 in the 5’UTR region, and 6 in the exonic region. When these variants were assessed based on their functional effects, 6 were found to be non-synonymous coding and 11 to be synonymous coding. This distribution indicates that most variants are located outside protein-coding sequences, suggesting a potential contribution of non-coding and regulatory regions to HR variation, rather than direct alterations to protein structure. When a single variant was annotated to more than one gene, such as SNPs located at the boundaries between adjacent genes (referred to as “gene 2” and “gene 3” in the Supporting information), all associated genes were included in the analysis (S3A–S3C Table).

Quantile-quantile (Q-Q) plots for male and female larvae were used to evaluate the distribution of observed GWAS p-values relative to the expected distribution under the null hypothesis (S3A, S3B, S3D, S3E Fig). The deviation of the upper tail from the expected line under both control (standard medium) and salt-supplemented conditions suggests that a subset of associated variants may represent biological signals rather than random noise. Consistent with this interpretation, several genes selected for validation were located near variants showing stronger-than-expected statistical signals. Although some candidate genes were associated with variants showing only moderate statistical support, previous GWAS studies of complex traits indicate that such loci may still be biologically meaningful, particularly when supported by independent functional evidence [40]. In this context, some SNPs identified in our study were considered worthy of further evaluation and functional validation despite their limited statistical support, because they were consistent with the predicted functions of the affected genes and showed biologically relevant effect sizes. Therefore, these variants are treated here as candidate variants rather than definitive associations.

To identify over-represented biological pathways and assess the functional relevance of the GWAS candidate genes, gene set enrichment analysis (GSEA) was performed using the PANGEA web tool, based on hypergeometric testing with FDR correction [41,42]. While few terms remained significant after correction for multiple testing, an examination of terms ranked by nominal p-values should be interpreted with caution but nevertheless suggested a biologically coherent pattern for HR (S3E Table). To evaluate the complex and evolutionarily conserved genetic mechanisms underlying the HR phenotype, GO enrichment analyses were performed using the identified candidate genes and their human orthologs. Functional annotation of the candidate genes revealed nominal enrichment in processes such as heart and muscle development (e.g., GO:0007507, GO:0055007, GO:0060562), ion transmembrane transport (GO:0034220), and signaling pathways regulating heart homeostasis (e.g., GO:0007186, GO:0000165, GO:0048010). Furthermore, gene-disease association analyses indicated potential links between these candidates and cardiovascular-related phenotypes such as hypertension, heart failure, and QT interval abnormalities. Given that gene set enrichment analyses identify over-represented biological patterns rather than provide definitive statistical evidence, these results were interpreted as suggestive and used to guide candidate gene prioritization. Candidate genes were selected by integrating biological insights from enrichment analyses with statistical evidence, including sex-specific p-values and variant effect sizes, and were subsequently prioritized for RNAi-based functional validation.

### Functional assessment

To validate the functional roles of candidate genes identified by the GWAS as being associated with HR, we performed in vivo analyses using the UAS/GAL4 system. RNAi lines used in these experiments were derived from two independent VDRC libraries (KK and GD), representing distinct insertion strategies and genetic backgrounds, allowing independent validation of gene-specific effects. While *Mef2-*GAL4 targets cardiac muscle cells and the contraction mechanism, *Hand*-GAL4 is expressed in broader cardiac and pericardial cell populations. By comparing these drivers, we were able to distinguish between structural contributions to cardiac organization and cell-type-specific regulatory effects on HR. Validation analyses were performed using two distinct statistical approaches. Initially, an analysis of variance (ANOVA) was performed for each candidate gene, considering control genotype (genetic background), treatment, and sex as variables, followed by Bonferroni-corrected post-hoc comparisons (S2B Table). Second, Dunnett’s test was used to compare all candidate genes to a single control genotype, with sexes being examined separately. Finally, the results from both methods were compared to make sure they were identical.

Experiments using the *Mef2* driver revealed that 20 candidate genes showed statistically significant differences in female larvae reared on standard medium, as confirmed by both statistical tests (Fig 3A, 3B). This represents 69% of the 29 genes analyzed (S5B Table). The candidate genes *tup* and *Rootletin* exhibited statistically significant differences only in the post-hoc tests following ANOVA, whereas results from Dunnett’s test did not confirm these findings. Furthermore, among the candidate genes validated in females, RNAi-mediated knockdown of 16 genes resulted in an increased HR relative to controls, whereas 4 genes caused a significant reduction. Validation of candidate genes in female larvae reared on salt-supplemented medium also identified 20 genes with statistically significant differences across both tests (S6A, S6B Table). Notably, we observed environment-specific effects: the *sns* gene, which increased HR in standard medium, caused no significant change under salt stress (S4A, S4B Fig). Conversely, the *MSI* gene, which showed no effect in standard medium, significantly increased HR relative to the control group in salt-reared larvae (S4E, S4F Fig).

**Fig 3:**
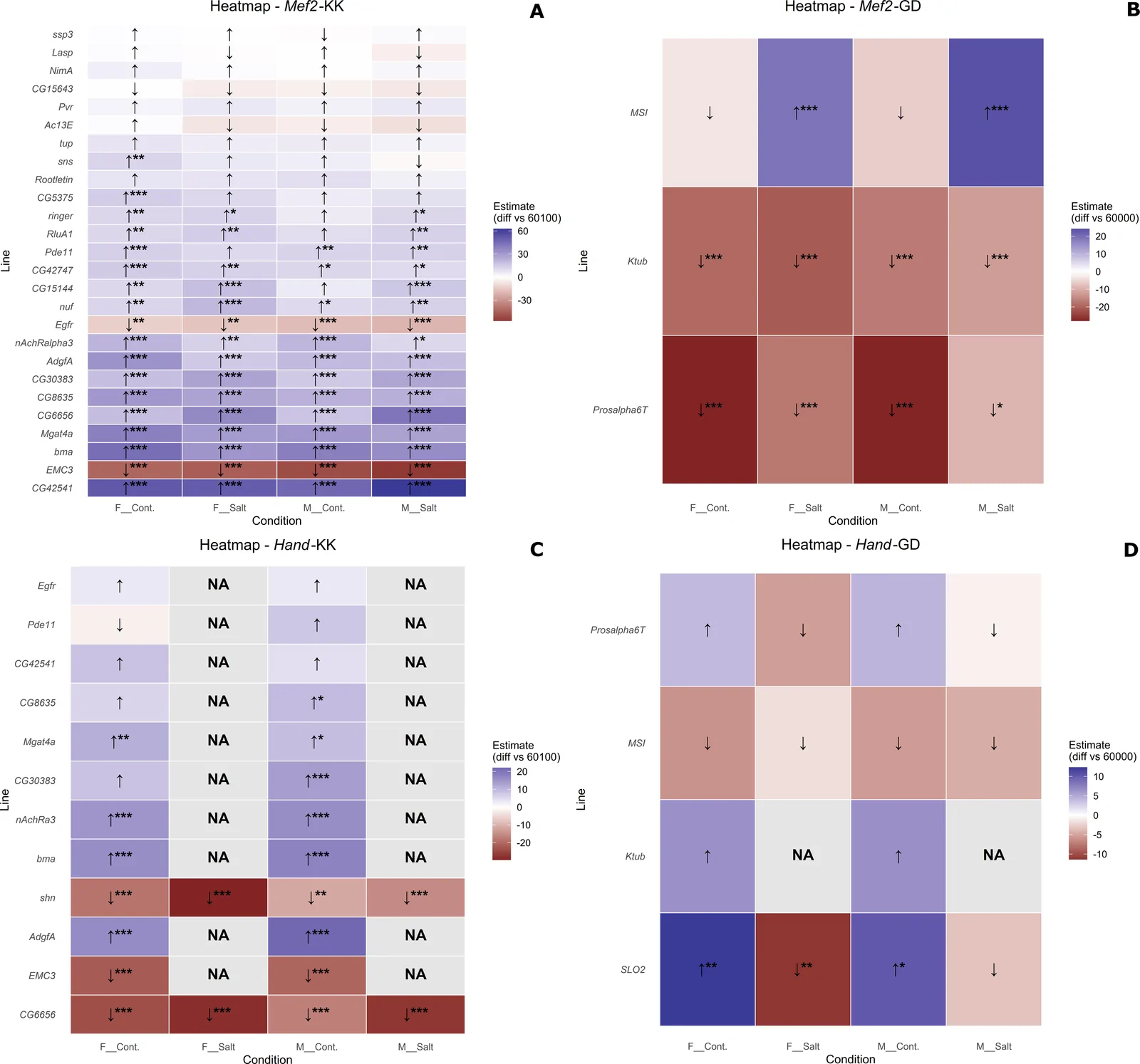
Heatmaps illustrate changes in heart rate (HR) across KK and GD RNAi lines driven by Mef2 and Hand. The color palette represents increases (positive values) and decreases (negative values) in HR. Arrows indicate the direction of change, asterisks denote levels of statistical significance (* P < 0.05, ** P < 0.01, *** P < 0.001), and NA indicates combinations for which no measurements were available. **(A)** Mef2–KK RNAi lines, **(B)** Mef2–GD RNAi lines, **(C)** Hand–KK RNAi lines, and **(D)** Hand–GD RNAi lines. Columns represent control (Cont.) and salt-supplemented (Salt) conditions in female and male individuals. KK and GD indicate independent Vienna Drosophila Resource Center (VDRC) RNAi libraries. Control comparisons were performed against the corresponding genetic background control lines VDRC 60100 (KK) and VDRC 60000 (GD). Additional validation analyses using the VDRC 60101 control line and independent GD RNAi backgrounds are presented in S6A–S6F Fig and S7A–S7F Fig.

In male larvae reared on standard medium, RNAi-mediated knockdown experiments revealed statistically significant differences in 15 candidate genes compared to the control genotype, as confirmed by both statistical tests. For the five genes out of these 15 (*CG15144*, *CG5375*, *RluA-1*, *ringer*, *sns*), we observed sex-specific effects; while validated in females reared on standard medium, these genes did not show statistically significant impact on HR in males. In addition, of the 15 genes identified to modulate HR in males, silencing of 11 resulted in elevated HRs, while 4 led to a significant reduction relative to the control genotype (S6C Table, S4C, S4G Fig). Following RNAi knockdown in male larvae reared on salt-containing medium, a total of 19 genes were determined to be statistically significant by both validation methods (S6D Table, S4D, S4H Fig). Specifically, four genes (*CG15144, RluA-1, ringer, MSI*) exhibited environment-dependent effects; while they showed no significant impact on HR in standard medium, they resulted in a significant increase relative to the control genotype in the salt-supplemented environment.

The 14 candidate genes validated in both sexes and by both statistical tests using the *Mef2* driver were subsequently examined using the *Hand* driver (Fig 3C, 3D). In female larvae reared on standard medium, *Hand*-mediated knockdown resulted in significant HR changes for 7 genes (*Adgf-A, CG42541, CG6656, EMC3, Mgat4a, bma,* and *nAChRα3*), as confirmed by both statistical approaches. Specifically, 5 of these genes increased HR, whereas silencing of *CG6656* and *EMC3* caused a reduction (S6E, S6G Table, S5A Fig). Male larvae exhibited a comparable pattern, with a total of 7 genes (*Adgf-A, CG30383, CG6656, EMC3, Mgat4a, bma, nAChRα3*) showing statistically significant differences relative to control genotype. The validated gene set was identical to that of females, except for one candidate. Consistent with the observations in females, knockdown of *CG6656* and *EMC3* reduced HR relative to the control genotype, whereas the remaining 5 genes caused an increase (S6F, S6H Table, S5C Fig). Additionally, the *SLO2* and *shn* genes did not produce viable offspring when crossed with the *Mef2* driver, but when crossed with the *Hand* driver, they resulted in statistically significant changes in HR in both media (S6 Table, S5 Fig).

In addition to HR phenotypes, developmental viability outcomes were noted during RNAi validation experiments. While most RNAi-driver combinations produced viable progeny, several crosses exhibited failure to eclose or absence of viable offspring (S4 Table). These observations were not collected as part of a formal survival assay and therefore were not subjected to quantitative analysis; however, they may indicate broader developmental roles for some of the identified candidate genes.

### Assessment of potential 40D insertion-associated effects

Previous studies have demonstrated that a subset of VDRC KK RNAi lines carrying the 40D insertion site may exhibit unintended phenotypic effects due to ectopic expression of the neighboring gene *tiptop* (*tio*), potentially confounding the interpretation of RNAi-induced phenotypes [43,44]. Therefore, candidate genes represented by KK RNAi lines known or suspected to carry the 40D construct were further evaluated using both the VDRC 60101 control line and independent GD RNAi lines targeting the same genes. This approach was adopted to assess whether the observed phenotypes were robust across alternative control backgrounds and independent RNAi constructs, consistent with recommendations emphasizing validation using independent RNAi reagents [45].

The additional analyses focused exclusively on the subset of candidate genes identified in the KK RNAi collection that were selected for independent validation because of the potential confounding effects associated with the KK 40D insertion site. In the *Mef2* driver background, all re-tested candidates remained statistically significant in the GD collection relative to their corresponding controls, as previously observed in the KK background. However, only *EMC3* retained the same direction of effect, whereas the remaining candidates exhibited opposite directional responses (S8A–S8D Table, S6A–S6F Fig). In the *Hand* driver background, statistical significance relative to the independent KK control (60101) was retained for only a subset of the re-tested candidates, whereas comparisons using GD RNAi lines supported significant effects for most candidates (S8A–S8D Table, S7A–S7F Fig). Overall, these findings indicate that the reproducibility of candidate gene effects depended on both the RNAi genetic background and the GAL4 driver. Therefore, both statistical significance and the direction of the phenotypic response should be considered when interpreting the validation results [46]. These results indicate that the direction and, in some cases, the magnitude of phenotypic responses varied across the validation conditions tested. Therefore, interpretation of individual phenotypes should take into account the RNAi background (KK and GD) and GAL4 driver context (*Mef2* and *Hand*) used in each experiment.

## Discussion

Cardiovascular diseases are among the leading causes of mortality worldwide, and fundamental physiological parameters, such as HR, play a crucial role in the pathogenesis of these diseases [1,3]. Several epidemiological and genomic studies have demonstrated a significant association between an increased resting HR and an elevated risk of cardiovascular disease and overall mortality [4–6]. Nevertheless, HR is a polygenic trait affected by multiple genes and may demonstrate considerable variation due to environmental and physiological influences [12]. This indicates that HR is a complex phenotype characterized by inadequately understood genetic factors and environmental stress responses [10]. *Drosophila melanogaster*, which has evolutionarily conserved cardiac development and electrical activity, is a powerful model for investigating the genetic architecture of this complex phenotype [14,15].

In this study, 154 DGRP lines were raised in different environments (standard medium and medium with 0.1% NaCl) to investigate genetic variation and genotype-environment interactions, with HR measured in L3 larvae. Our findings revealed that larval HR is a quantitative trait with a strong genetic basis that varies according to environmental conditions. Differences of up to approximately threefold were observed between the lines in both environments, and this variation was supported by moderate broad-sense heritability values (*H^2^ ≈* 0.5). Remarkably, the slight increase in broad-sense heritability estimates observed under salt stress, coupled with the distinct lack of overlap in GWAS candidates between standard and salt-supplemented conditions, suggests a release of cryptic genetic variation (CGV). By definition, CGV refers to standing genetic variation that remains phenotypically silent under standard physiological conditions but contributes to phenotypic variance when the organism is challenged by environmental perturbation. This finding underscores the critical role of genotype-by-environment (GxE) interactions in unmasking hidden genetic components of cardiac regulation that remain undetectable under standard conditions. These observed moderate broad-sense heritability estimates strongly support a polygenic architecture, in which multiple loci with small to moderate effect sizes shape the HR phenotype. These findings are correlated with those from other complex phenotypes studied via the DGRP, suggesting that HR is significantly affected by natural genetic variation [34,47].

Research on larval HR has generally been concentrated on a limited number of genotypes through optogenetic, pharmacological, or environmental changes [12,48–50]. Even though these studies clearly show that specific gene mutations and environmental variables such as temperature or calcium levels impact HR, they fail to account for natural genetic variation, gene interactions, and genotype-environment interactions. Conversely, our study is among the first to comprehensively examine the genetic basis of larval HR variation as a component of cardiac physiology across a broad panel of natural variation (154 DGRP lines). Our findings reveal that the effects of environmental variables on HR depend on genetic background, providing new and unique insights into the natural genetic architecture of this complex phenotype.

To better understand the potential functional mechanisms underlying natural genetic variation in HR, selected candidate genes identified through genome-wide association studies (GWAS) were targeted using RNA interference (RNAi). Considering that these candidate genes may exert distinct functions across different cell types, the selected candidates were crossed with two different driver lines. This approach enabled a coarse spatial dissection of gene function, allowing us to infer in which cellular populations gene knockdown impacts HR.

Candidate genes that cause significant changes in HR when knocked down in both *Mef2* and *Hand* backgrounds may be important for how the heart functions. Given the partially overlapping but distinct cellular targets of these drivers, similar phenotypes suggest a comprehensive regulatory function, encompassing both the structural domains delineated by *Hand* expression and the functional cardiomyocyte populations influenced by *Mef2*. Conversely, candidates validated exclusively through the *Mef2* driver seem to operate via cardiomyocyte-intrinsic mechanisms, including ion homeostasis and cellular equilibrium, a conclusion that is consistent with their previously delineated molecular functions.

Results from RNAi validation experiments indicate that the confirmed candidate genes contribute to HR regulation and are associated with distinct functional categories. Gene Ontology analyses aimed at determining the functional classifications of these genes highlighted key biological pathways linked to cardiac function and homeostasis. The findings indicate that the majority of genes are classified into fundamental categories, including developmental regulation, ion balance, electrical activity, protein folding and quality control, metabolic regulation, and cellular stress responses. This multi-layered functional architecture reveals that HR is a complex physiological trait governed by the interplay of multiple evolutionarily conserved pathways and the extensive network of genes involved, rather than being controlled by a single pathway.

Importantly, these functional categories are not independent but are mechanistically interconnected within cardiac cells. Ion channels regulate the electrical excitability of the heart, while protein quality control systems ensure the correct folding, trafficking, and membrane localization of these channels. In parallel, metabolic pathways and stress-response mechanisms modulate cellular energy availability and intracellular signaling, thereby influencing ion channel activity and contractile performance. This integrative framework suggests that HR variation arises from the coordinated interplay of electrophysiological, proteostatic, and metabolic processes rather than from isolated gene effects.

The *shn* gene is excluded from these main groups because it is associated with heart development. The knockdown of the *shn* gene via *Hand*-GAL4 crossing, which is associated with epithelial tube morphogenesis (GO:0060562) during heart development, resulted in a significant decrease in HR in *Drosophila* [51,52]. *shn* is an important factor that controls the *tinman* gene, which is necessary for heart development in the dorsal mesoderm, via the *Dpp* signaling pathway [53]. The evolutionary conservation of the *HIVEP* gene family in humans and the association of *HIVEP3* with left ventricular hypertrophy in a GWAS study suggest that *shn* may be one of the important genes involved in regulating HR [54]. Notably, among the 14 functionally validated candidate genes discussed in this study, nine (*shn, bma, SLO2, CG42541, CG6656, Prosalpha6T, Ktub, Adgf-A,* and *Egfr*) possess human orthologs that have previously been associated with cardiovascular disease phenotypes, including cardiomyopathies, cardiac hypertrophy, arrhythmias, congenital heart defects, heart failure, cardiac dysfunction, and myocardial abnormalities. The remaining five genes (*nAChRα3, Mgat4a, EMC3, Adgf-A2,* and *CG8635*) are indirectly linked to cardiovascular biology through pathways related to autonomic regulation, ion-channel modulation, vascular integrity, adenosine metabolism, inflammatory signaling, and cardiac remodeling. Together, these observations support the potential translational relevance of the identified candidate genes and are consistent with the evolutionary conservation of molecular pathways contributing to cardiac function between *Drosophila* and humans.

However, because a subset of VDRC KK RNAi lines has been reported to exhibit insertion-associated phenotypic effects due to ectopic expression of *tiptop* (*tio*), additional validation experiments were performed using the VDRC 60101 control line and independent GD RNAi lines for candidate genes represented by KK lines carrying or suspected to carry the 40D construct [43,44]. These findings demonstrated that the core phenotypic pattern was largely preserved among the re-tested candidate genes, with most candidates remaining significantly different from their corresponding controls across the validation experiments. The observed variation was driven primarily by shifts in the direction of the phenotypic response rather than by the loss of statistical significance, highlighting the influence of RNAi background and GAL4 driver context on individual gene effects. Therefore, although the identified candidates collectively implicate multiple biological pathways in HR regulation, the level of support for individual genes varies across the validation conditions tested. Accordingly, gene-specific phenotypic effects should be interpreted in the context of the RNAi background and driver used.

### RNAi validation highlights genes associated with ionic regulation of heart rate

Our RNAi validation experiments have identified several genes that play a role in ionic homeostasis, which is important for regulating HR. The precise movement of ions, such as sodium, potassium, and calcium, across the cardiac cell membrane regulates the formation of action potentials and the rhythmic contraction-relaxation cycle of the heart muscle. Imbalances in this process can disrupt impulse conduction and cause arrhythmia. Therefore, maintaining proper ion homeostasis and electrical activity is essential for preserving the HR. In this context, *nAChRα3*, *CG42541*, *Mgat4a*, *bma* and *SLO2* have been found to be associated with mechanisms regulating the heart’s ion balance and electrical activity of the heart [48,55–59]. When silenced in both *Mef2* and *Hand* driver lines, these genes also showed statistically significant (p < 0.001) phenotypic differences compared to the controls.

Knockdown of the *nAChRα3* gene via RNAi led to a significant increase in HR. Although nicotinic acetylcholine receptors in *Drosophila* are primarily found in the central nervous system, the activation of these receptors by acetylcholine and its agonists, nicotine and muscarine, has been reported to increase the HR [48]. Furthermore, this effect has been attributed to a localized increase in intracellular calcium release by acetylcholine receptors located in the endoplasmic reticulum membrane. In humans, the orthologs of this gene, *CHRNA2* and *CHRNA3*, also encode nicotinic receptors and increase the HR by weakening parasympathetic signal transmission through decreased gene expression [58].

In parallel, the knockdown of the *bma* gene induced a striking change relative to the control, resulting in a significant increase in HR. While this gene is known to play a role in nociception mechanisms, its cardiac physiological function remains unknown [60]. However, the human ortholog of this gene, *SCYL2*, is involved in regulating voltage-gated calcium channels and exerts a protective effect against cell death (excitotoxicity) associated with calcium overload. *SCYL2* deficiency in mice has been reported to cause epileptic seizures and congenital heart anomalies, suggesting that the gene establishes a critical link between calcium metabolism and cardiac function [61,62]. Taken together, these observations suggest that *bma* may influence HR through pathways associated with ion homeostasis and calcium metabolism.

Also, a significant increase in HR has been observed as a result of the specific knockdown of the *SLO2* gene in cardiac tissue. Through controlling the activity of sodium-activated potassium channels in both humans and *Drosophila*, the *SLO2* gene contributes to the suppression of hyperexcitability in response to elevated intracellular sodium [55]. According to earlier research, these channels help control HR by shortening the action potential in mammalian cardiomyocytes [56]. Additionally, the results of this study are in line with the correlation between QT prolongation and arrhythmia susceptibility and mutations in the human ortholog *KCNT1* gene, which implies that the *SLO2* gene is one of the genes involved in HR regulation [57].

Besides these candidates, *Mgat4a* knockdown led to a significant rise in HR relative to the control, even though no prior relationship with *Drosophila* heart physiology has been documented. However, the human ortholog *MGAT4D* plays an indirect role in regulating calcium channels via glycosylation, acting as an inhibitor of the *MGAT1* enzyme [59,63]. Based on the reported role of *MGAT4D* in calcium channel regulation and glycosylation-related processes, *Mgat4a* may influence HR through effects on ion-channel regulation and cellular ion homeostasis.

One of the most striking findings of this study is that, among all validated candidate genes, *CG42541* exhibited the most profound effect on HR; the knockdown of this gene resulted in an increase of 52 beats per 20 seconds relative to the control. It is associated with GTP binding and calcium channel regulation in *Drosophila*. The human orthologsof this gene are *REM1*, *REM2*, and *GEM*. These RGK-related proteins prevent the functioning of voltage-gated calcium channels [64,65]. Heart conduction abnormalities, such as Timothy syndrome and long QT syndrome, are known to result from mutations in these proteins [64]. Therefore, it is believed that this gene plays a role in controlling cardiac physiology and that the increase in HR observed when *CG42541* is knocked down is caused by an excessive contraction response induced by increased intracellular calcium accumulation. Taken together, these findings show that ion balance plays a fundamental role in regulating HR, both in terms of electrical conduction and in maintaining cellular integrity and the contraction cycle. The candidate genes validated in our study affect different levels of calcium metabolism and channel activity, which demonstrates that ion homeostasis in cardiac physiology is under multi-component genetic control.

Building on these gene-specific observations, a unifying mechanistic framework emerges in which multiple candidate genes converge on shared electrophysiological processes.

At the mechanistic level, these findings indicate that multiple candidate genes converge on calcium-dependent excitation–contraction coupling, a central process that links electrical activity to mechanical contraction. Perturbations in ion channel regulation, calcium handling, or membrane excitability are therefore likely to produce coordinated changes in HR by altering action potential dynamics and intracellular calcium flux. Because these channels must first be correctly synthesized, folded, and trafficked to the membrane before they can support such activity, we next considered the proteostatic machinery underlying their biogenesis.

### Genes associated with protein homeostasis contribute to heart rate

Consistent with the proteostatic component of this framework, three of the validated candidate genes - *EMC3*, *CG6656*, and *Prosalpha6T* - act on the machinery that folds, processes, and degrades ion-channel proteins rather than on the channels themselves. The *EMC3* gene, which was found to have one of the most noticeable phenotypic effects in the study, reduced the HR by approximately 50 beats per 20 s compared to the control. *EMC3* is a major component of the endoplasmic reticulum membrane complex in both *Drosophila* and humans. It functions as an insertase, ensuring the correct insertion of transmembrane proteins into the membrane and plays a role in the biosynthesis of voltage-gated ion channels [66]. It has also been reported to play an important role in vascular integrity and development [67]. Although *EMC3* has not been directly linked to cardiac physiology previously, the substantial decrease in HR noted following *EMC3* knockdown supports a potential role for *EMC3* in cardiac activity, particularly through pathways related to membrane protein biogenesis and proteostasis.

The *CG6656* gene exhibited opposite phenotypes in the two driver lines; it increased the HR in the *Mef2* driver but decreased it in the *Hand* driver. This gene, which has been reported in *Drosophila* databases to regulate acid phosphatase activity, has no direct relationship with cardiac physiology. The human ortholog *ACP2* encodes the lysosomal acid phosphatase enzyme and is involved in protein quality control [68]. Lysosomal dysfunction leads to protein aggregation and the development of associated cardiomyopathy. These contrasting effects suggest that the increase in *Mef2* driver reflects the systemic stress response, whereas the decrease in *Hand* driver reflects the loss of lysosomal function in heart cells. These discrepancies may arise from the two drivers beginning to manifest in distinct cells and at varying developmental periods. Therefore, *CG6656* can be considered an important candidate gene associated with lysosomal quality control pathways in cardiac physiology.

*Prosalpha6T* shows a significant phenotype only when the *Mef2* driver is used, and it encodes a subunit of the 26S proteasome complex. There have been no reported direct associations with heart physiology in *Drosophila*. The human ortholog *PSMA1* is a key component of the ubiquitin–proteasome system that plays a critical role in protein quality control. *PSMA1* deficiency leads to cellular stress and heart failure associated with misfolded protein accumulation [69]. Consequently, the decrease in HR observed when *Prosalpha6T* is silenced can be attributed to reduced proteasomal activity [70]. Because both channel biogenesis and its quality-control machinery are energetically costly processes, we next asked whether metabolic and stress-response pathways shape heart rate through a comparable logic.

### Genes involved in metabolic balance and stress response contribute to heart rate variation

Two of the validated candidates, *ktub* and *Adgf*-A, act within thismetabolic and regulatory context, linking cellular energy homeostasis and receptor-mediated signaling to cardiac output.

In the case of *Ktub* gene, which is knocked-down by the *Mef2* driver, results showed a significant decrease in HR which was associated with the “regulation of G protein-coupled receptor-mediated signaling” process in Gene Ontology analyses. The literature shows that neurotransmitters such as serotonin, dopamine, and octopamine strongly modulate *Drosophila* heart rhythm via G protein-coupled receptors (GPCRs) [71]. We propose that the reduction in HR following *ktub* knockdown results from disrupted GPCR signaling pathways, causing impaired ion flow and reduced electrical activity. The human ortholog *TULP3* functions as a GPCR carrier protein, and mutations in this gene have been reported to disrupt the WNT and TGF-β pathways, affecting cardiac structural integrity and being associated with hypertrophic cardiomyopathy [72]. This suggests that the *ktub* gene may have an evolutionarily conserved regulatory role in maintaining cardiac function via GPCR-mediated signaling.

One important enzyme involved in adenosine degradation is encoded by the candidate gene, *Adgf-A*. Knockdown of the gene encoding this enzyme with both drivers resulted in a highly significant elevation in HR. Adenosine accumulation, excessive receptor stimulation, and a subsequent systemic stress response are the outcomes of the absence of this gene in *Drosophila* [73]. Similarly, the human ortholog *ADA2* controls adenosine metabolism; its overexpression results in developmental abnormalities, whereas its deficiency compromises endothelial integrity and causes cardiac dysfunction [74]. Therefore, the increase in HR observed with *Adgf-A* silencing may be a secondary reflection of systemic stress associated with adenosine accumulation.

*Adgf-A2* (*MSI*), a member of the same gene family, demonstrated significant differences only under salt-containing conditions and when using the *Mef2* driver. As *Adgf* genes are known to compensate for each other, the observed increase in HR in *Adgf-A2* deficiency is believed to be a compensatory response to environmental stress [75]. These results imply a close relationship between adenosine metabolism, energy balance, stress response, and cardiac adaptation.

In addition to *Adgf-A2*, *CG8635* and *Egfr* genes have been found to be associated with cellular stress responses and signal transduction processes. The roles of these genes in cardiac physiology can be evaluated within the framework of adaptive responses that develop under stressful conditions.

The *Mef2* driver silencing of the *CG8635* gene was shown to significantly increase the HR; however, this gene has not yet been directly connected to cardiac physiology. The human ortholog *ZC3H15* has primarily been characterized in cancer-related studies, where it has been found to play a role in regulating the TNF receptor NF-κB signaling pathway by interacting with *TRAF2* [76]. This interaction plays a crucial role in myocardial fibrosis, inflammation, cell survival and regeneration, and cardiac tissue remodeling by controlling the expression of genes that are part of the NF-κB cascade [77,78]. Therefore, the increase in HR observed with *CG8635* silencing can be considered a secondary effect of disrupting NF-κB-mediated signaling. The potential function of this gene requires further investigation in basic and translational models.

However, the *Egfr* gene caused a significant decrease in HR only when knocked down with the *Mef2* driver. The epidermal growth factor receptor (*EGFR*) is a key signaling component involved in the maintenance of cardiac development and function in both *Drosophila* and humans [79]. *EGFR*-mediated receptor tyrosine kinase (RTK) signaling is essential for the survival of cardiac mesoderm cells and maintenance of myocardial integrity [80]. The decrease in HR observed upon *EGFR* silencing supports the direct role of this gene in maintaining ion balance and sustaining electrical activity. This finding further demonstrates that *EGFR* signaling is a central regulator of evolutionarily conserved cardiac networks.

It is important to note that the findings of this study are specific to the larval stage, as the *Drosophila* heart undergoes significant remodeling during metamorphosis [38,39]. In adults, additional structural features and neuronal regulation contribute to more complex cardiac dynamics, including heartbeat reversal [15,38]. These developmental differences are likely to influence HR regulation and should be considered when extrapolating our findings across developmental stages [38].

The functional relationships among the validated candidate genes and the major biological processes identified in this study are summarized in Fig 4.

**Fig 4:**
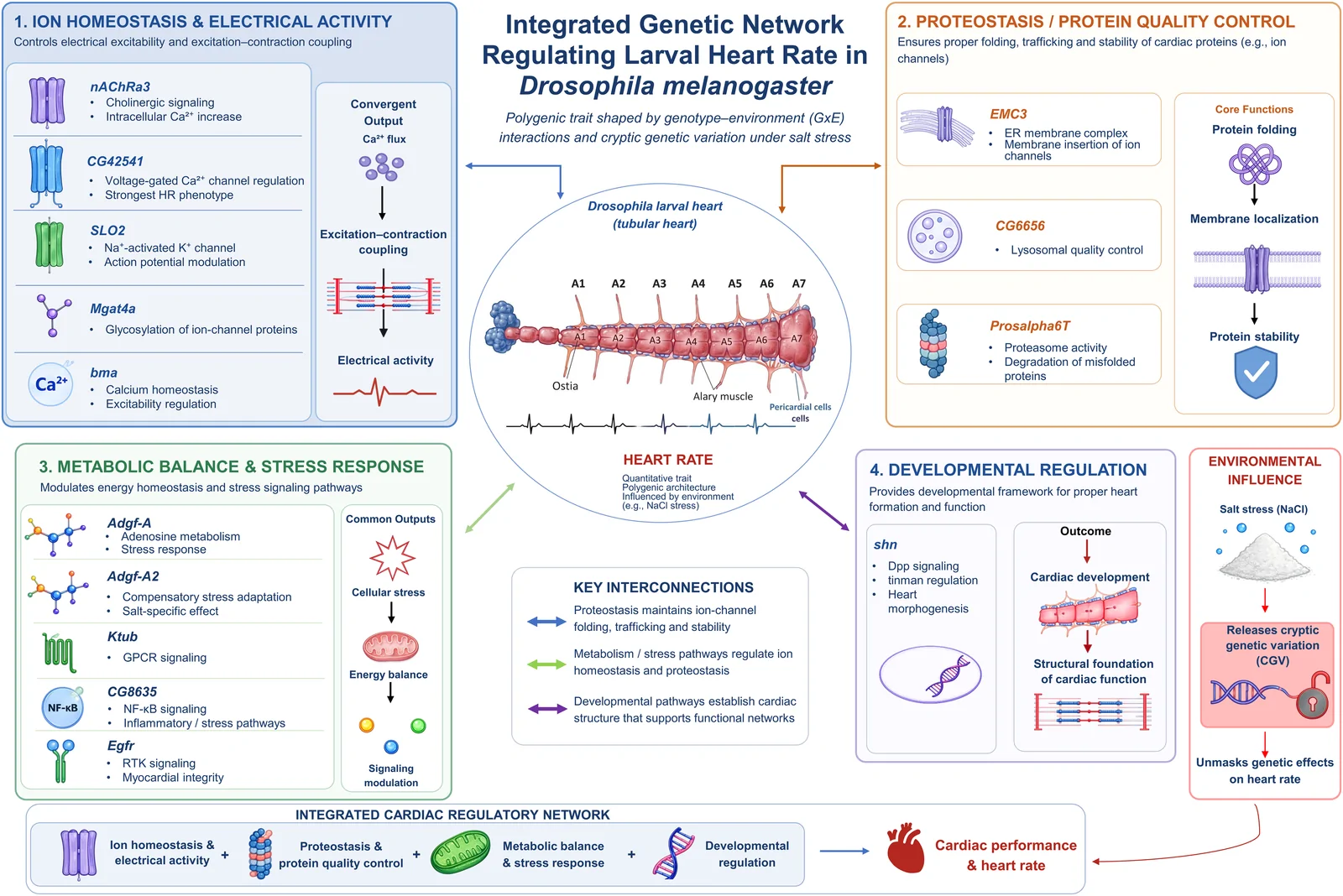
Conceptual model of genetic architecture underlying HR regulation in *Drosophila* larvae. The model integrates GWAS findings and RNAi validation results, illustrating how candidate genes converge on interconnected pathways involving ion homeostasis, proteostasis, metabolism, stress response, and developmental regulation. These pathways collectively influence cardiac performance and contribute to genotype- and environment-dependent variation in HR. Arrows represent proposed functional relationships inferred from gene annotations, Gene Ontology enrichment analyses, and published literature. The illustrative clip-art elements in this figure were generated using the AI image-generation tool in ChatGPT (OpenAI), from text prompts created by the authors; the overall figure design, arrangement, and scientific content were produced by the authors.

Taken together, our findings support a system-level model of cardiac regulation in which multiple genetic modules, including ion homeostasis, proteostasis, metabolism, and stress signaling-interact to determine cardiac performance. Rather than acting independently, these modules form an integrated network that governs cardiomyocyte function and physiological output. In this context, HR emerges as a complex phenotype shaped by the coordinated interplay of these interconnected pathways, rather than by isolated gene effects. Our findings demonstrate that cardiac regulation is orchestrated by evolutionarily conserved genetic networks common to both *Drosophila* and humans [15,39]. This integrative framework is consistent with the evolutionary conservation of core cardiac regulatory mechanisms between species.

This conservation is particularly evident in core processes such as excitation–contraction coupling, calcium signaling, and stress-response pathways, which operate through homologous genes and regulatory networks in both *Drosophila* and human cardiomyocytes. Accordingly, even subtle alterations in these networks can precipitate profound functional impairments, effectively recapitulating the genetic basis of human cardiac pathologies.

The RNAi-based approach employed in this study provided functional evidence supporting the involvement of multiple candidate genes in HR regulation. Future studies integrating protein interaction networks, tissue-specific expression analyses, and cell-type-specific approaches will further enhance our understanding of the molecular mechanisms underlying the observed phenotypic effects.

Additional research should be conducted to investigate the role of these genetic factors in regulating heart rhythm using vertebrate models, integrated with calcium dynamics analyses, and translational studies in human-derived cardiomyocytes. This study highlights the importance of understanding the genetic architecture of HR through analyses carried out in a broad genetic background that includes natural variation and genotype x environment interactions. Within this framework, the *Drosophila* model is an excellent experimental tool for understanding this complicated system at many levels.

## Materials & methods

### *Drosophila* stocks and husbandry

Fly lines were obtained from the Bloomington *Drosophila* Stock Center and the Vienna *Drosophila* Resource Center. All stocks were maintained on standard *Drosophila* food [81] at a temperature of 25°C, with a relative humidity of 60–75%, and under a 12-hour light–dark cycle. The group subjected to salt-supplementation was reared in a standard culture medium containing 0.1% NaCl. For HR imaging, 20 females and 20 males at the third larval stage (L3) were used from each of the 154 DGRP lines and treatment groups. This design allowed us to partition genetic, environmental, and sex-specific effects on HR variation. To perform the experiments, the DGRP lines were randomly selected. In the validation experiments, 20 females and 20 males at the L3 larval stage from each cross (RNAi-mediated gene expression-reduced crosses) and treatment group were used. In addition, qualitative observations regarding progeny viability (viable offspring, failure to eclose, or absence of viable progeny) were recorded during RNAi validation experiments. A list of the lines used in the validation experiments and viability status of RNAi crosses is provided in the Supporting information (RNAi lines list is provided in S4 Table).

### Heart-specific RNAi gene expression silencing in *Drosophila*

Tissue-specific gene knockdown was achieved using the binary UAS/GAL4 expression system in combination with RNA interference (RNAi) [80]. In this system, a tissue-specific driver line expresses the yeast transcription factor GAL4, which binds Upstream Activating Sequences (UAS) to activate a downstream UAS-linked transgene exclusively in the target tissue; when this transgene encodes a gene-specific RNAi construct, it triggers sequence-specific degradation of the corresponding transcript, thereby silencing the gene of interest in that tissue [82]. GAL4 drivers specific to the *Mef2* and *Hand* genes, which play critical roles in heart development, were used in crosses [17,83–85]. Unmated females from the driver lines were crossed with males from the UAS lines to reduce heart-specific gene expression. RNAi lines were obtained from the Vienna Drosophila Resource Center (VDRC) and consisted of two independent libraries: the KK collection, which contains site-specific insertions at a defined genomic locus, and the GD collection, which includes randomly inserted RNAi constructs. RNAi lines in both collections are generated in defined genetic backgrounds, allowing controlled comparisons across lines. Corresponding control lines (VDRC60100 for KK and VDRC60000 for GD) were used as genetic background controls in all comparisons. In addition, because the 40D insertion present in a subset of VDRC KK RNAi lines has been reported to induce ectopic *tiptop* (*tio*) expression, potentially confounding phenotype interpretation, selected candidate genes were re-evaluated using the VDRC60101 control line and independent GD RNAi lines targeting the same genes [43,44]. This approach was employed to assess the reproducibility of the observed phenotypes across alternative control backgrounds and independent RNAi backgrounds and served as an additional quality-control measure during phenotype interpretation. Heartbeat recordings were performed on L3 larvae from the offspring of these crosses. This approach enables functional assessment of candidate genes at the phenotypic level by evaluating the effects of reduced gene expression on HR.

### Preparation, sex identification and imaging of larvae for heart rate measurements

Flies from each DGRP line and validation cross studied were allowed to lay eggs in standard medium and in a medium containing 0.1% NaCl. The L3 larvae were collected on the day of the experiment. The larvae were identified as male or female using a Leica M205C stereo microscope. Sex was identified based on the presence or absence of a transparent oval structure visible in male larvae representing the gonads [86]. The larvae were separated by sex and fixed onto transparent tape with a water-based adhesive, with their abdominal sections facing the adhesive surface. Excess water was absorbed with blotting paper, and HR videos were recorded using a Flexacam C3 camera mounted on a Leica M205C stereo microscope. After the HR recordings were completed, each larva was transferred individually to tubes containing standard *Drosophila* culture medium and maintained until eclosion. Sex determination was subsequently verified after eclosion.

### Quantification and statistical analyses

All videos obtained for each line, treatment group, and sex were slowed down using the VLC player to obtain HR data. Each larval video was 20 seconds long. Data were obtained by counting the total number of contractions in each larval video recording (Raw data are provided in S1A Table; normality tests are provided in S7 Table). All HR measurements were evaluated based on the number of contractions recorded during a fixed observation period of 20 s. The data were not converted to a per-minute basis, and all experimental groups were analyzed using the same time window.

Although several automated software platforms are available for *Drosophila* cardiac phenotyping, many of these systems require high-frame-rate recordings (commonly ≥100 fps) for accurate analysis of cardiac parameters [83]. Since the Flexacam C3 camera used in this study recorded videos at 30 fps, these software requirements could not be adequately met under our experimental conditions. Therefore, HR measurements were performed manually by a single observer using a standardized scoring procedure to minimize observer-related variability.

HR data were quantified from video recordings and subsequently analyzed using a combination of descriptive statistics, mixed-effects modeling, and additional statistical tests (including Welch’s t-tests and multiple testing corrections) to assess genetic, environmental, and sex-specific effects.

A mixed-effects model was used for variance analysis to explain the phenotypic variability of the HR. The model included sex (S) and treatment (T; control or salt diet) as fixed effects, as well as the interaction between these two terms (T x S) in the model. DGRP lines (L) and two-way interactions between line and fixed effects (L × S and L × T) were included as random effect components. Genotype-by-environment (G × E) interactions were quantified using the Line × Treatment interaction term in the mixed-effects model, which captures differences in phenotypic responses of genotypes across environmental conditions. A significant interaction indicates that the effect of the environment on HR varies depending on genetic background.

Reaction norms were used as a complementary conceptual framework to visualize these interactions, where each line represents the phenotypic response of a given genotype across environments (S1A and S1B Fig). Variation in the slope and direction of these lines reflects genotype-specific sensitivity to environmental change.

Mathematically, the model is expressed as follows:


Y = μ + S + T +(T×S) +L +(L×S) + (L×T) + ε

Here, Y represents the phenotype, μ represents the overall mean, and ε represents the residual variance of the model. Additionally, analyses were repeated using reduced models fitted separately for each treatment and sex. In these reduced models, treatment, sex, and their interaction terms were removed by analyzing each treatment–sex combination independently. Broad-sense heritability (*H²*) was estimated to quantify the proportion of total phenotypic variance attributable to genetic differences among DGRP lines, based on variance components derived from the mixed-effects model. Variance components were calculated for all random effects, and the broad-sense heritability (*H²*) for HR in L3 larvae obtained from DGRP lines for each application group was calculated separately for each sex and application as follows:


$$\begin{array}{c} \;H^{\text{2}}=\frac{\sigma_{L}^{\text{2}}}{\sigma_{L}^{\text{2}}+\;\sigma_{\varepsilon }^{\text{2}}} \end{array}$$

where σ²_*L*_ represents the variance among DGRP lines (genetic variance component) and σ²_*ε*_ corresponds to the residual (error) variance. This estimate reflects the proportion of phenotypic variance attributable to genetic differences among lines. To complement the population-level variance estimates obtained from the mixed-effects model, line-specific comparisons were additionally performed to identify genotypes showing differential responses to salt exposure. Welch’s t-tests were performed for each Line × Sex combination to evaluate differences in HR between larvae developed on standard and salt-supplemented media. Resulting p-values were adjusted separately for females and males using the Benjamini–Hochberg false discovery rate (FDR) procedure to control multiple testing across lines.

Data obtained from HR measurements conducted on F1 larvae derived from UAS/GAL4 crosses in the validation experiments were subjected to a median absolute deviation (MAD)-based outlier removal method to minimize the impact of outliers on the analysis. MAD was chosen because it robustly captures the dispersion of the distribution, has low sensitivity to outliers, and provides the best fit to the sample size and data structure (Raw data from RNAi crosses are provided in S5A Table). The dataset, which was made more compatible with parametric assumptions by removing outliers using this method, was analyzed using Dunnett’s test. This test was used to compare each RNAi line to its corresponding control background while controlling for multiple comparisons, allowing for a more statistically consistent comparison of multiple genes with the same control group (using VDRC60100 and VDRC60000 as background-matched control lines for the KK and GD libraries, respectively).

All data analyses were performed using the R programming language [87]. The R scripts used for statistical analyses and figure generation are provided as S1 Code to facilitate reproducibility.

### Genome-wide association and gene set enrichment analyses

GWAS analyses were conducted on line means, as averaging across individuals within each inbred line minimizes environmental and stochastic variation, thereby enriching the phenotypic signal for heritable genetic differences among lines. Genome-wide association analyses were conducted using a two-step linear mixed model-based framework adapted for DGRP data. Prior to association testing, phenotypes were adjusted for environmental and technical confounders using a pre-trained correction model applied separately to female, male, average, and sexual dimorphism (female minus male) traits.

Variants were filtered based on minor allele frequency (MAF ≥ 0.05), call rate (≥ 0.80), and a minimum minor allele count of 4, with a minimum of 30 lines required per variant. Following these filtering steps, a total of 1,900,106 variants were retained and tested for association across 154 DGRP lines. Association testing was carried out using PLINK for initial screening, followed by a linear mixed model (LMM) approach implemented in FaST-LMM, in which a pre-computed genomic relatedness matrix (GRM) was included as a random effect to account for population structure and genetic relatedness among inbred lines.

The association between the SNPs identified in each DGRP line and relevant phenotypes was tested using a mixed-model approach. Each of the four phenotypic traits - female, male, average, and sexual dimorphism - was tested independently. Hypothesis testing was applied to these variants, and polymorphisms exceeding the p < 10^5^ threshold were identified as candidate variants at a suggestive significance level, consistent with previous GWAS studies conducted in *Drosophila melanogaster* inbred line panels. These candidate SNPs, insertions, and deletions pointed to gene regions that could contribute to the investigated phenotype at the genetic level, enabling the identification of potential genetic determinants [35,88,89]. Top variants were subsequently annotated with gene and regulatory region information.

Linkage disequilibrium among candidate variants was assessed and visualized as a heatmap, and quantile-quantile (Q-Q) plots were generated to evaluate potential departure from the null distribution of test statistics. GWAS analyses were performed using data on the mean HR values of larvae reared in control and salt-supplemented media and the mean difference between the two groups. The complete GWAS analysis pipeline is provided as S2 Code to ensure reproducibility of the analyses.

To investigate the functional properties of the candidate genes identified through GWAS, gene set enrichment analyses (GSEA) were conducted using the Pathway, Network, and Gene-set Enrichment Analysis (PANGEA) database [41,42]. This approach enables the identification of over-represented biological processes and pathways by comparing the gene list against curated gene sets from multiple annotation databases, including Gene Ontology (GO), pathway databases, and disease association resources. Within the PANGEA platform, statistical enrichment is evaluated using hypergeometric testing with false discovery rate (FDR) correction to identify over-represented gene sets.

Candidate genes were selected for validation experiments based on the multifaceted information obtained from these analyses. P-values and effect sizes associated with variants were considered based on annotation data from GWAS outputs, and gene set enrichment results were used to provide functional context, including biological processes, potential disease associations, and information on human orthologs. Given that enrichment analyses identify patterns of over-representation rather than direct causal relationships, these results were interpreted as suggestive and used as an exploratory framework for candidate prioritization. Consequently, the validation studies included both statistically robust candidates and biologically meaningful genes with the potential to contribute to clinical research.

## Supporting information

S1 Table DGRP raw data and descriptive statistics. Raw heart rate measurements and descriptive statistics for the 154 DGRP lines under control and salt-supplemented conditions.
(XLSX)

S2 Table Analysis of variance (ANOVA) results. ANOVA outputs for the DGRP lines and for the RNAi validation experiments.
(XLSX)

S3 Table GWAS annotations. Top-variant annotations, candidate genes, and Gene Ontology terms identified in the genome-wide association analyses.
(XLSX)

S4 Table RNAi line list and progeny viability status. List of RNAi lines used in the validation experiments and the viability status of each cross.
(XLSX)

S5 Table RNAi cross raw data and descriptive statistics. Raw heart rate data and descriptive statistics for the RNAi validation crosses.
(XLSX)

S6 Table Dunnett’s test versus ANOVA comparison. Comparison of the results obtained from Dunnett’s test and from ANOVA for the RNAi validation experiments.
(DOCX)

S7 Table Normality test results. Normality tests for the DGRP larval heart rate data.
(DOCX)

S8 Table Additional GD RNAi line experiments. Raw data, descriptive statistics, ANOVAs, and Dunnett’s test results for the additional experiments assessing candidate genes with independent GD RNAi lines and the VDRC 60101 control (40D insertion-associated controls).
(XLSX)

S1 Fig Reaction norm plots. Reaction norms illustrating genotype-by-environment interactions in heart rate across DGRP lines.
(DOCX)

S2 Fig Cross-environment correlation graphs. Correlations of line-mean heart rate between standard and salt-supplemented conditions.
(DOCX)

S3 Fig Q-Q plots, Manhattan plots, and linkage disequilibrium (LD) heatmaps. Diagnostic and association plots from the genome-wide association analyses.
(DOCX)

S4 Fig RNAi knockdown boxplots (*Mef2* driver). Heart rate distributions for candidate-gene knockdowns using the *Mef2* driver.
(DOCX)

S5 Fig RNAi knockdown boxplots (*Hand* driver). Heart rate distributions for candidate-gene knockdowns using the *Hand* driver.
(DOCX)

S6 Fig Additional validation experiments (*Mef2* driver). Comparisons of *Mef2*-driven candidate-gene knockdowns between KK and GD RNAi lines and against the VDRC 60101 control, used to assess 40D insertion-associated effects.
(DOCX)

S7 Fig Additional validation experiments (*Hand* driver). Comparisons of *Hand*-driven candidate-gene knockdowns between KK and GD RNAi lines and against the VDRC 60101 control, used to assess 40D insertion-associated effects.
(DOCX)

S1 Code Statistical analyses and figure generation. R scripts used to perform the statistical analyses (descriptive statistics, ANOVA, mixed-effects models, Welch’s t-tests, and the RNAi validation tests) and to generate the figures.
(R)

S2 Code GWAS analysis pipeline. R scripts implementing the genome-wide association analysis pipeline, including variant filtering and the FaST-LMM mixed-model association testing.
(R)
