# Supplementary material for: Genetic architecture of basal heart rate and its modulation by dietary salt in Drosophila melanogaster
Source: PLoS Genet. 2026 Oct 5;22(10):e1012313. doi: 10.1371/journal.pgen.1012313 (PMC13637896; doi:10.1371/journal.pgen.1012313)
Supplement: Supplementary file 1 [file pgen.1012313.s006.docx]

**S6 Table.** Comparison of Dunnett test and ANOVA results for genes silenced via RNAi using *Mef2* and *Hand*-GAL4 drivers. (A) Female larvae in control medium (*Mef2* GAL4 driver). (B) Female larvae in salt-supplemented medium (*Mef2* GAL4 driver). (C) Male larvae in control medium (*Mef2* GAL4 driver). (D) Male larvae in salt-supplemented medium (*Mef2* GAL4 driver). (E) Female larvae in control medium (*Hand* GAL4 driver). (F) Male larvae in control medium (*Hand* GAL4 driver). (G) Female larvae in salt-supplemented medium (*Hand* GAL4 driver). (H) Male larvae in salt-supplemented medium (*Hand* GAL4 driver).

(A) Female larvae in control medium (*Mef2* GAL4 driver).

|  |  |  | **Post-hoc test results after ANOVA** | | | **Dunnett test results** | | |
| --- | --- | --- | --- | --- | --- | --- | --- | --- |
| **Gene** | **Control Genotype** | **Δ** | **t ratio** | **p value** | **Significance** | **t ratio** | **p value** | **Significance** |
| *Ac13E* | 60100 | 1,11 | 0,40 | 0,688 | - | 0,31 | 1,000 | - |
| *Adgf-A* |  | 32,5 | 10,75 | 0,000 | *** | 10,06 | 0,000 | *** |
| *CG15144* |  | 11,6 | 11,64 | 0,000 | *** | 3,50 | 0,011 | ** |
| *CG15643* |  | -0,43 | 1,89 | 0,819 | - | -0,12 | 1,000 | - |
| *CG30383* |  | 18,8 | 6,64 | 0,000 | *** | 5,53 | 0,000 | *** |
| *CG42541* |  | 52,2 | 13,8 | 0,000 | *** | 15,16 | 0,000 | *** |
| *CG42747* |  | 15,7 | 6,32 | 0,000 | *** | 4,56 | 0,000 | *** |
| *CG5375* |  | 14,7 | 4,86 | 0,000 | *** | 4,39 | 0,000 | *** |
| *CG6656* |  | 19,4 | 5,92 | 0,000 | *** | 5,39 | 0,000 | *** |
| *CG8635* |  | 31,3 | 10,5 | 0,000 | *** | 9,08 | 0,000 | *** |
| *EMC3* |  | -44,2 | 16,5 | 0,000 | *** | -9,76 | 0,000 | *** |
| *Egfr* |  | -13,4 | 4,75 | 0,000 | *** | -3,66 | 0,006 | ** |
| *Lasp* |  | 1,0 | 0,54 | 0,593 | - | 0,29 | 1,000 | - |
| *Mgat4a* |  | 38,9 | 14,9 | 0,000 | *** | 11,42 | 0,000 | *** |
| *NimA* |  | 4,9 | 1,78 | 0,076 | - | 1,38 | 0,860 | - |
| *Pde11* |  | 14,2 | 5,41 | 0,000 | *** | 4,17 | 0,001 | *** |
| *Pvr* |  | 3,6 | 1,56 | 0,120 | - | 1,04 | 0,965 | - |
| *RluA-1* |  | 12,0 | 4,33 | 0.000 | *** | 3,56 | 0,008 | ** |
| *Rootletin* |  | 8,0 | 2,96 | 0,003 | ** | 2,35 | 0,260 | - |
| *bma* |  | 44,7 | 13,0 | 0,000 | *** | 13,14 | 0,000 | *** |
| *nAChRalpha3* |  | 21,9 | 9,39 | 0,000 | *** | 6,28 | 0,000 | *** |
| *nuf* |  | 12,4 | 4,10 | 0,000 | *** | 3,54 | 0,009 | ** |
| *ringer* |  | 12,1 | 6,28 | 0,000 | *** | 3,46 | 0,012 | * |
| *sns* |  | 12,7 | 4,27 | 0,000 | *** | 3,59 | 0,008 | ** |
| *ssp3* |  | 0,8 | 0,31 | 0,749 | - | 0,22 | 1,000 | - |
| *tup* |  | 8,1 | 3,58 | 0,004 | ** | 2,33 | 0,273 | - |
| *ktub* | 60000 | -19,1 | 5,06 | 0,000 | *** | 5,91 | 0,000 | *** |
| *MSI* |  | -3,2 | 0,72 | 0,478 | - | 0,92 | 0,664 | - |
| *Prosalpha6T* |  | -27,9 | 6,95 | 0,000 | *** | 8,43 | 0,000 | *** |

(B) Female larvae in salt-supplemented medium (*Mef2* GAL4 driver).

|  |  |  | **Post-hoc test results after ANOVA** | | | **Dunnett test results** | | |
| --- | --- | --- | --- | --- | --- | --- | --- | --- |
| **Gene** | **Control Genotype** | **Δ** | **t ratio** | **p value** | **Significance** | **t ratio** | **p value** | **Significance** |
| *Ac13E* | 60100 | -7,4 | 2,7 | 0,008 | ** | -2,1 | 0,433 | - |
| *Adgf-A* |  | 15,9 | 4,9 | 0,000 | *** | 4,6 | 0,000 | *** |
| *CG15144* |  | 19,1 | 7,1 | 0,000 | *** | 5,3 | 0,000 | *** |
| *CG15643* |  | -5,6 | 2,8 | 0,005 | ** | -1,5 | 0,780 | - |
| *CG30383* |  | 27,4 | 9,0 | 0,000 | *** | 7,5 | 0,000 | *** |
| *CG42541* |  | 50,7 | 13,2 | 0,000 | *** | 14,4 | 0,000 | *** |
| *CG42747* |  | 13,7 | 5,3 | 0,000 | *** | 3,8 | 0,003 | ** |
| *CG5375* |  | 11,3 | 3,5 | 0,001 | *** | 3,2 | 0,031 | * |
| *CG6656* |  | 35,9 | 10,9 | 0,000 | *** | 10,0 | 0,000 | *** |
| *CG8635* |  | 27,7 | 9,1 | 0,000 | *** | 7,9 | 0,000 | *** |
| *EMC3* |  | -46,4 | 20,8 | 0,000 | *** | -12,3 | 0,000 | *** |
| *Egfr* |  | -16,4 | 4,9 | 0,000 | *** | -3,8 | 0,003 | ** |
| *Lasp* |  | -0,8 | 0,4 | 0,707 | - | -0,2 | 1,000 | - |
| *Mgat4a* |  | 30,2 | 11,3 | 0,000 | *** | 8,6 | 0,000 | *** |
| *NimA* |  | 1,3 | 0,5 | 0,642 | - | 0,4 | 1,000 | - |
| *Pde11* |  | 11,3 | 4,2 | 0,000 | *** | 3,2 | 0,026 | * |
| *Pvr* |  | 6,9 | 2,9 | 0,004 | ** | 1,9 | 0,531 | - |
| *RluA-1* |  | 14,6 | 5,0 | 0,000 | *** | 4,1 | 0,001 | *** |
| *Rootletin* |  | 8,1 | 2,9 | 0,005 | ** | 2,3 | 0,307 | - |
| *bma* |  | 32,0 | 9,3 | 0,000 | *** | 9,4 | 0,000 | *** |
| *nAChRalpha3* |  | 13,8 | 5,7 | 0,000 | *** | 3,8 | 0,003 | ** |
| *nuf* |  | 21,1 | 7,3 | 0,000 | *** | 6,3 | 0,000 | *** |
| *ringer* |  | 13,1 | 6,5 | 0,000 | *** | 3,6 | 0,007 | ** |
| *sns* |  | 6,6 | 2,3 | 0,025 | * | 1,9 | 0,549 | - |
| *ssp3* |  | 0,3 | 0,12 | 0,910 | - | 0,1 | 1,000 | - |
| *tup* |  | 5,7 | 2,4 | 0,016 | * | 1,6 | 0,752 | - |
| *ktub* | 60000 | -20,9 | 5,5 | 0,000 | *** | 5,9 | 0,000 | *** |
| *MSI* |  | 18,9 | 4,4 | 0,000 | *** | 5,2 | 0,000 | *** |
| *Prosalpha6T* |  | -17,1 | 4,3 | 0,000 | *** | 4,8 | 0,000 | *** |

(C) Male larvae in control medium (*Mef2* GAL4 driver).

|  |  |  | **Post-hoc test results after ANOVA** | | | **Dunnett test results** | | |
| --- | --- | --- | --- | --- | --- | --- | --- | --- |
| **Gene** | **Control Genotype** | **Δ** | **t ratio** | **p value** | **Significance** | **t ratio** | **p value** | **Significance** |
| *Ac13E* | 60100 | -5,1 | 1,8 | 0,073 | - | -1,4 | 0,858 | - |
| *Adgf-A* |  | 20,7 | 5,7 | 0,000 | *** | 5,4 | 0,000 | *** |
| *CG15144* |  | 6,2 | 2,1 | 0,413 | - | 1,5 | 0,780 | - |
| *CG15643* |  | -4,1 | 2,2 | 0,027 | * | -1,2 | 0,925 | -- |
| *CG30383* |  | 15,9 | 5,4 | 0,000 | *** | 4,5 | 0,000 | *** |
| *CG42541* |  | 46,9 | 12,3 | 0,000 | *** | 13,5 | 0,000 | *** |
| *CG42747* |  | 12,0 | 4,6 | 0,000 | *** | 3,3 | 0,019 | * |
| *CG5375* |  | 5,7 | 1,8 | 0,071 | *** | 1,6 | 0,721 | - |
| *CG6656* |  | 17,4 | 5,4 | 0,000 | *** | 4,9 | 0,000 | *** |
| *CG8635* |  | 23,3 | 7,7 | 0,000 | *** | 6,6 | 0,000 | *** |
| *EMC3* |  | -51,5 | 26,8 | 0,000 | *** | -15,8 | 0,000 | *** |
| *Egfr* |  | -18,6 | 7.1 | 0,000 | *** | -5,5 | 0,000 | *** |
| *Lasp* |  | 0,4 | 0,2 | 0,816 | - | 0,1 | 1,000 | - |
| *Mgat4a* |  | 31,2 | 11,5 | 0,000 | *** | 8,8 | 0,000 | *** |
| *NimA* |  | 0,7 | 0,3 | 0,793 | - | 0,2 | 1,000 | - |
| *Pde11* |  | 12,8 | 4,7 | 0,000 | *** | 3,6 | 0,008 | ** |
| *Pvr* |  | 4,3 | 1,9 | 0,061 | - | 1,2 | 0,911 | - |
| *RluA-1* |  | 9,8 | 3,3 | 0,001 | *** | 2,7 | 0,110 | - |
| *Rootletin* |  | 9,4 | 3,4 | 0,001 | *** | 2,7 | 0,118 | - |
| *bma* |  | 38,9 | 10,8 | 0,000 | *** | 10,9 | 0,000 | *** |
| *nAChRalpha3* |  | 21,6 | 9,3 | 0,000 | *** | 6,2 | 0,000 | *** |
| *nuf* |  | 11,1 | 3,6 | 0,0003 | ** | 3,2 | 0,033 | * |
| *ringer* |  | 6,1 | 3,1 | 0,003 | ** | 1,7 | 0,684 | - |
| *sns* |  | 5,3 | 1,8 | 0,067 | - | 1,5 | 0,774 | - |
| *ssp3* |  | -0,7 | 0,3 | 0,077 | - | -0,2 | 1,000 | - |
| *tup* |  | 6,0 | 2,6 | 0,01 | * | 1,7 | 0,690 | - |
| *ktub* | 60000 | -16,7 | 4,5 | 0,000 | *** | 4,1 | 0,000 | *** |
| *MSI* |  | 6,1 | 1,5 | 0,134 | - | 1,5 | 0,309 | - |
| *Prosalpha6T* |  | -27,7 | 7,2 | 0,000 | *** | 6,8 | 0,000 | *** |

(D) Male larvae in salt-supplemented medium (*Mef2* GAL4 driver).

|  |  |  | **Post-hoc test results after ANOVA** | | | **Dunnett test results** | | |
| --- | --- | --- | --- | --- | --- | --- | --- | --- |
| **Gene** | **Control Genotype** | **Δ** | **t ratio** | **p value** | **Significance** | **t ratio** | **p value** | **Significance** |
| *Ac13E* | 60100 | -8,9 | 3.3 | 0,001 | *** | -2,5 | 0,171 | - |
| *Adgf-A* |  | 19,3 | 5,9 | 0,000 | *** | 5,5 | 0,000 | *** |
| *CG15144* |  | 17,0 | 6,5 | 0,000 | *** | 4,9 | 0,000 | *** |
| *CG15643* |  | -6,6 | 3,7 | 0,0003 | ** | -2,0 | 0,485 | - |
| *CG30383* |  | 26,6 | 9,4 | 0,000 | *** | 7,8 | 0,000 | *** |
| *CG42541* |  | 62,8 | 16,8 | 0,000 | *** | 18,4 | 0,000 | *** |
| *CG42747* |  | 10,7 | 4,3 | 0,000 | *** | 3,1 | 0,039 | * |
| *CG5375* |  | 9,1 | 2,8 | 0,005 | ** | 2,6 | 0,157 | - |
| *CG6656* |  | 42,3 | 13,8 | 0,000 | *** | 12,6 | 0,000 | *** |
| *CG8635* |  | 23,6 | 7,9 | 0,000 | *** | 6,9 | 0,000 | *** |
| *EMC3* |  | -57,1 | 28,9 | 0,000 | *** | -17,2 | 0,000 | *** |
| *Egfr* |  | -20,5 | 7,8 | 0,000 | *** | -6,0 | 0,000 | *** |
| *Lasp* |  | -4,7 | 2,5 | 0,013 | * | -1,4 | 0,867 | - |
| *Mgat4a* |  | 27,9 | 10,1 | 0,000 | *** | 7,7 | 0,000 | *** |
| *NimA* |  | 2,7 | 1,0 | 0,312 | - | 0,8 | 0,993 | - |
| *Pde11* |  | 13,4 | 4,9 | 0,000 | *** | 3,8 | 0,003 | ** |
| *Pvr* |  | 6,9 | 2,9 | 0,003 | ** | 2,0 | 0,488 | - |
| *RluA-1* |  | 13,7 | 4,7 | 0,000 | *** | 3,9 | 0,002 | ** |
| *Rootletin* |  | 4,7 | 1,7 | 0,09 | - | 1,3 | 0,872 | - |
| *bma* |  | 34,9 | 9,1 | 0,000 | *** | 9,2 | 0,000 | *** |
| *nAChRalpha3* |  | 11,7 | 5,1 | 0,000 | *** | 3,4 | 0,014 | * |
| *nuf* |  | 13,7 | 4,3 | 0,000 | *** | 3,7 | 0,005 | ** |
| *ringer* |  | 12,1 | 6,3 | 0,000 | *** | 3,5 | 0,012 | * |
| *sns* |  | -2,0 | 0,7 | 0,483 | - | -0,6 | 0,999 | - |
| *ssp3* |  | 1,7 | 0,7 | 0,484 | - | 0,5 | 1,000 | - |
| *tup* |  | 3,7 | 1,7 | 0,09 | - | 1,1 | 0,956 | - |
| *ktub* | 60000 | -12,3 | 3,4 | 0,0009 | *** | 4,1 | 0,0003 | *** |
| *MSI* |  | 24,2 | 6,1 | 0,000 | *** | 8,1 | 0,000 | *** |
| *Prosalpha6T* |  | -9,1 | 2,3 | 0,023 | * | 2,9 | 0,013 | * |

(E) Female larvae in control medium (*Hand* GAL4 driver).

|  |  |  | **Post-hoc test results after ANOVA** | | | **Dunnett test results** | | |
| --- | --- | --- | --- | --- | --- | --- | --- | --- |
| **Gene** | **Control Genotype** | **Δ** | **t ratio** | **p value** | **Significance** | **t ratio** | **p value** | **Significance** |
| *Adgf-A* | 60100 | 16,7 | -4,676 | 0,000 | *** | 5,57 | 0,000 | *** |
| *CG30383* |  | 8,4 | -2,435 | 0,0170 | ** | 2,63 | 0,069 | - |
| *CG42541* |  | 8,53 | -2,593 | 0,0112 | * | 2,78 | 0,046 | * |
| *CG6656* |  | -23,9 | 8,268 | 0,000 | *** | -6,67 | 0,000 | *** |
| *CG8635* |  | 5,86 | -1,800 | 0,075 | . | 1,91 | 0,32 | - |
| *EMC3* |  | -22,3 | 6,724 | 0,000 | *** | -7,08 | 0,000 | *** |
| *Egfr* |  | 3,32 | -0,973 | 0,33 | - | 1,09 | 0,82 | - |
| *Mgat4a* |  | -1,3 | -3,210 | 0,0019 | ** | 3,48 | 0,0015 | ** |
| *Pde11* |  | -2,10 | 0,590 | 0,5566 | - | -0,67 | 0,96 | - |
| *bma* |  | 16,2 | -4,671 | 0,000 | *** | 5,21 | 0,000 | *** |
| *nAChRalpha3* |  | 15,1 | -4,796 | 0,000 | *** | 4,93 | 0,000 | *** |
| *shn* |  | -18,8 | 4,42 | 0,000 | *** | -4,98 | 0,000 | *** |
| *ktub* | 60000 | 6,59 | -2,153 | 0,0342 | . | 1,98 | 0,054 | - |
| *MSI* |  | -6,05 | 1,626 | 0,1057 | . | -1,64 | 0,23 | - |
| *Prosalpha6T* |  | 4,058 | -1,382 | 0,1689 | - | 1,16 | 0,46 | - |
| *SLO2* |  | 12,33 | -3,90 | 0,000 | *** | 3,48 | 0,002 | ** |

(F) Male larvae in control medium (*Hand* GAL4 driver).

|  |  |  | **Post-hoc test results after ANOVA** | | | **Dunnett test results** | | |
| --- | --- | --- | --- | --- | --- | --- | --- | --- |
| **Gene** | **Control Genotype** | **Δ** | **t ratio** | **p value** | **Significance** | **t ratio** | **p value** | **Significance** |
| *Adgf-A* | 60100 | 22,1 | -6,226 | 0,000 | *** | 7,21 | 0,000 | *** |
| *CG30383* |  | 13,9 | -4,328 | 0,000 | *** | 4,55 | 0,000 | *** |
| *CG42541* |  | 4,44 | -1,369 | 0,17 | - | 1,43 | 0,62 | - |
| *CG6656* |  | -17,0 | 5,659 | 0,000 | *** | -5,31 | 0,000 | *** |
| *CG8635* |  | 9,59 | -2,945 | 0,0042 | * | 3,05 | 0,021 | , |
| *EMC3* |  | -20,9 | 6,908 | 0,000 | *** | -7,08 | 0,000 | *** |
| *Egfr* |  | 3,16 | -0,940 | 0,35 | - | 1,03 | 0,85 | - |
| *Mgat4a* |  | 9,1 | -2,948 | 0,0041 | ** | 3,12 | 0,017 | * |
| *Pde11* |  | 7,84 | -2,392 | 0,0189 | * | 2,62 | 0,07 | - |
| *bma* |  | 17,9 | -5,463 | 0,000 | *** | 5,93 | 0,000 | *** |
| *nAChRalpha3* |  | 16,2 | -5,21 | 0,000 | *** | 5,24 | 0,000 | *** |
| *shn* |  | -11,6 | 2,76 | 0,006 | ** | -3,61 | 0,001 | ** |
| *ktub* | 60000 | 6,63 | -2,319 | 0,0228 | . | 2,52 | 0,015 | * |
| *MSI* |  | -5,53 | 1,671 | 0,0965 | . | -1,7 | 0,18 | - |
| *Prosalpha6T* |  | 4,380 | -1,517 | 0,1313 | - | 1,28 | 0,36 | - |
| *SLO2* |  | 10,23 | -3,53 | 0,001 | ** | 2,99 | 0,01 | * |

(G) Female larvae in salt-supplemented medium (*Hand* GAL4 driver).

|  |  |  | **Post-hoc test results after ANOVA** | | | **Dunnett test results** | | |
| --- | --- | --- | --- | --- | --- | --- | --- | --- |
| **Gene** | **Control Genotype** | **Δ** | **t ratio** | **p value** | **Significance** | **t ratio** | **p value** | **Significance** |
| *CG6656* | 60100 | -28,4 | 9,515 | 0,000 | *** | -6,37 | 0,000 | *** |
| *shn* |  | -29,66 | 7,14 | 0,000 | *** | -6,65 | 0,000 | *** |
| *MSI* | 60000 | -1,85 | 0,551 | 0,5827 | - | -0,608 | 0,69 | - |
| *Prosalpha6T* |  | -5,346 | 1,748 | 0,0824 | . | -1,60 | 0,12 | - |
| *SLO2* |  | -11,4 | 3,71 | 0,000 | *** | -3,604 | 0,001 | ** |

(H) Male larvae in salt-supplemented medium (*Hand* GAL4 driver).

|  |  |  | **Post-hoc test results after ANOVA** | | | **Dunnett test results** | | |
| --- | --- | --- | --- | --- | --- | --- | --- | --- |
| **Gene** | **Control Genotype** | **Δ** | **t ratio** | **p value** | **Significance** | **t ratio** | **p value** | **Significance** |
| *CG6656* | 60100 | -27,4 | 9,780 | 0,000 | *** | -9,07 | 0,000 | *** |
| *shn* |  | -15,6 | 3,87 | 0,000 | *** | -5,01 | 0,000 | *** |
| *MSI* | 60000 | -4,53 | 1,267 | 0,2068 | - | -1,58 | 0,254 | - |
| *Prosalpha6T* |  | 0,769 | 0,268 | 0,7892 | - | -0,28 | 0,94 | - |
| *SLO2* |  | -3,33 | 1,06 | 0,29 | - | -1,16 | 0,5 | - |
