# Supplementary material for: Genetic architecture of basal heart rate and its modulation by dietary salt in Drosophila melanogaster
Source: PLoS Genet. 2026 Oct 5;22(10):e1012313. doi: 10.1371/journal.pgen.1012313 (PMC13637896; doi:10.1371/journal.pgen.1012313)
Supplement: Supplementary file 2 [file pgen.1012313.s007.docx]

**S7 Table.** Normality tests. Prior to analysis, the data were subjected to outlier detection based on standardized residual values (rstand) obtained from the mixed-effects model used in the variance analysis. Data points showing significant deviation from the expected variance structure (rstand > 2 or rstand < −2) were defined as outliers and removed from the dataset.

Subsequently, normality tests were performed to assess the distribution of the heart rate phenotype and to ensure the validity of statistical analyses, including ANOVA and GWAS. For this purpose, the nortest and stats packages in R were used, providing Anderson–Darling, Cramer–von Mises, Lilliefors (Kolmogorov–Smirnov), Pearson Chi-Square, Shapiro–Francia, and Shapiro–Wilk tests.

The results indicate that different sex and treatment groups exhibit varying levels of deviation from normality; however, these deviations did not affect the robustness of subsequent analyses, consistent with previous findings (Schielzeth et al., 2020).

P-values for each test are shown for control (standard medium) and salt-supplemented conditions in female and male larvae.

| Tests | Control Female  (Pval) | Control Male (Pval) | Salt Female  (Pval) | Salt Male  (Pval) |
| --- | --- | --- | --- | --- |
| Anderson–Darling | 0,05353 | 0,3002 | 0,2489 | 0,2728 |
| Cramer–von Mises | 0,04697 | 0,332 | 0,3424 | 0,3247 |
| Lilliefors (KS) | 0,012 | 0,2507 | 0,541 | 0,4999 |
| Pearson Chi-Square | 0,6817 | 0,3316 | 0,6478 | 0,3872 |
| Shapiro–Francia | 0,09035 | 0,3063 | 0,002006 | 0,0004484 |
| Shapiro–Wilk | 0,1112 | 0,3526 | 0,00466 | 0,0007493 |

Schielzeth, H., Dingemanse, N. J., Nakagawa, S., Westneat, D. F., Allegue, H., Teplitsky, C., . . . Araya‐Ajoy, Y. G. (2020). Robustness of linear mixed‐effects models to violations of distributional assumptions. *Methods in ecology and evolution, 11*(9), 1141-1152.
