# Supplementary material for: Genetic architecture of basal heart rate and its modulation by dietary salt in Drosophila melanogaster
Source: PLoS Genet. 2026 Oct 5;22(10):e1012313. doi: 10.1371/journal.pgen.1012313 (PMC13637896; doi:10.1371/journal.pgen.1012313)
Supplement: Supplementary file 3 [file pgen.1012313.s009.docx]

**S1 Fig.** Reaction norm plots of heart rate across control (standard) and salt-supplemented conditions for DGRP lines measured at the third larval stage (L3). (A) Female larvae. (B) Male larvae. Each line represents the mean heart rate of a single DGRP line measured under control and salt conditions, illustrating the response of each genotype to environmental change. Variation in the slopes and directions of these lines indicates genotype-by-environment (G×E) interactions. The black line represents the overall mean response across all lines.
