# Supplementary material for: Genetic architecture of basal heart rate and its modulation by dietary salt in Drosophila melanogaster
Source: PLoS Genet. 2026 Oct 5;22(10):e1012313. doi: 10.1371/journal.pgen.1012313 (PMC13637896; doi:10.1371/journal.pgen.1012313)
Supplement: Supplementary file 4 [file pgen.1012313.s010.docx]

**S2 Fig.** Phenotypic correlations of heart rate between standard and salt-supplemented conditions in L3 larvae across 154 DGRP lines. (A) Female larvae. (B) Male larvae. Each point represents the mean heart rate of a DGRP line measured under control (x-axis) and salt (y-axis) conditions. The solid line indicates the linear regression fit, and the dashed line represents the identity line (y = x). Deviations from the identity line reflect genotype-specific responses to environmental change, consistent with genotype-by-environment interactions.
