# Supplementary material for: Genetic architecture of basal heart rate and its modulation by dietary salt in Drosophila melanogaster
Source: PLoS Genet. 2026 Oct 5;22(10):e1012313. doi: 10.1371/journal.pgen.1012313 (PMC13637896; doi:10.1371/journal.pgen.1012313)
Supplement: Supplementary file 5 [file pgen.1012313.s011.docx]

**S3 Fig.** P-value distributions and genome-wide linkage disequilibrium (LD) patterns derived from GWAS analyses. All distributions correspond to females, males, and sex-averaged phenotypes, as these analyses yielded the highest number of candidate variants.

**(A–F)** Quantile–quantile (Q–Q) plots showing the distribution of observed versus expected p-values under the null hypothesis. (A) Female, standard medium; (B) Male, standard medium; (C) Sex average, standard medium; (D) Female, salt-supplemented medium; (E) Male, salt-supplemented medium; (F) Sex average, salt-supplemented medium.

**(G–H)** Genomic locations of variants exceeding the suggestive significance threshold (P < 1×10⁻⁵). The five major *Drosophila* chromosomes are shown along the x-axis. (G) Standard medium; (H) Salt-supplemented medium.

**(I–J)** Genome-wide linkage disequilibrium (LD) heatmaps illustrating pairwise correlations among candidate variants. (I) Standard medium; (J) Salt-supplemented medium.
