# Supplementary material for: Genetic architecture of basal heart rate and its modulation by dietary salt in Drosophila melanogaster
Source: PLoS Genet. 2026 Oct 5;22(10):e1012313. doi: 10.1371/journal.pgen.1012313 (PMC13637896; doi:10.1371/journal.pgen.1012313)
Supplement: Supplementary file 7 [file pgen.1012313.s013.docx]

**S5 Fig.** Heart rate (HR) variation across RNAi lines with heart-specific gene expression reduction driven by the *Hand*-GAL4 system under standard and salt-supplemented conditions. Boxplots show HR measurements for female and male larvae in KK and GD RNAi backgrounds. Individual data points represent biological replicates.

**(A–D)** KK RNAi lines: (A) female larvae under standard conditions, (B) female larvae under salt-supplemented conditions, (C) male larvae under standard conditions, and (D) male larvae under salt-supplemented conditions.

**(E–H)** GD RNAi lines: (E) female larvae under standard conditions, (F) female larvae under salt-supplemented conditions, (G) male larvae under standard conditions, and (H) male larvae under salt-supplemented conditions.

**Asterisks indicate statistically significant differences relative to the corresponding genetic background control line (Dunnett’s test; *P < 0.05, **P < 0.01, ***P < 0.001).
