# Supplementary material for: Genetic architecture of basal heart rate and its modulation by dietary salt in Drosophila melanogaster
Source: PLoS Genet. 2026 Oct 5;22(10):e1012313. doi: 10.1371/journal.pgen.1012313 (PMC13637896; doi:10.1371/journal.pgen.1012313)
Supplement: Supplementary file 9 [file pgen.1012313.s015.docx]

**S7 Fig.** Heart rate comparisons of Hand-GAL4-driven RNAi lines in KK and GD collections.

(A–B) Comparisons between KK RNAi lines and the original genetic background control line (60100). (C–D) Comparisons between the same KK RNAi lines and an independent control line (60101). (E–F) Comparisons between GD RNAi lines and the corresponding genetic background control line (60000). Significant differences from the respective control lines are indicated by asterisks. The persistence of significant effects in comparisons with both 60100 and 60101 suggests that the observed KK phenotypes are unlikely to result solely from control-line-specific effects. Most candidate genes exhibited consistent directional effects across KK and GD collections, providing additional support for their contribution to heart rate regulation. However, genes displaying divergent responses between RNAi collections should be interpreted with caution. **Asterisks indicate statistically significant differences relative to the corresponding genetic background control line (Dunnett’s test; *P < 0.05, **P < 0.01, ***P < 0.001).
